# Genotypic and phenotypic analyses of a *Pseudomonas aeruginosa* chronic bronchiectasis isolate reveal differences from cystic fibrosis and laboratory strains

**DOI:** 10.1186/s12864-015-2069-0

**Published:** 2015-10-30

**Authors:** John J. Varga, Mariette Barbier, Xavier Mulet, Piotr Bielecki, Jennifer A. Bartell, Joshua P. Owings, Inmaculada Martinez-Ramos, Lauren E. Hittle, Michael R. Davis, F. Heath Damron, George W. Liechti, Jacek Puchałka, Vitor A. P. Martins dos Santos, Robert K. Ernst, Jason A. Papin, Sebastian Albertí, Antonio Oliver, Joanna B. Goldberg

**Affiliations:** Department of Pediatrics, Division of Pulmonology, Allergy/Immunology, Cystic Fibrosis and Sleep, Children’s Healthcare of Atlanta, Atlanta, GA USA; Emory + Children’s Center for Cystic Fibrosis Research, Emory University and Children’s Healthcare of Atlanta, Atlanta, GA USA; Department of Microbiology, Immunology, and Cancer Biology, University of Virginia, Charlottesville, VA USA; Department of Microbiology, Immunology and Cell Biology, West Virginia University School of Medicine, Morgantown, WV USA; Servicio de Microbiología and Unidad de Investigación, Hospital Son Espases, Instituto de Investigación Sanitaria de Palma (IdISPa), Palma, de Mallorca Spain; Synthetic and Systems Biology Research Group, Helmholtz Centre for Infection Research, Braunschweig, Germany; Department of Biomedical Engineering, University of Virginia, Charlottesville, VA USA; IUNICS, University of the Balearic Islands, Palma, de Mallorca Spain; Department of Microbial Pathogenesis, University of Maryland School of Dentistry, University of Maryland, Baltimore, MD USA; Systems and Synthetic Biology, Wageningen University, Wageningen, Netherlands; Present address: Immunobiology Department, Yale University, School of Medicine, New Haven, CT 06511 USA; Present address: Dr. von Hauner Children’s Hospital, Ludwig Maximilians University, Munich, Germany; Present address: Chair of Systems and Synthetic Biology, Wageningen University, Wageningen, The Netherlands; Present address: LifeGlimmer GmbH, Berlin, Germany

**Keywords:** *Pseudomonas aeruginosa*, Metabolic model, Transcriptome, Comparative genomics, Cystic fibrosis, Bronchiectasis

## Abstract

**Background:**

*Pseudomonas aeruginosa* is an environmentally ubiquitous Gram-negative bacterium and important opportunistic human pathogen, causing severe chronic respiratory infections in patients with underlying conditions such as cystic fibrosis (CF) or bronchiectasis. In order to identify mechanisms responsible for adaptation during bronchiectasis infections, a bronchiectasis isolate, PAHM4, was phenotypically and genotypically characterized.

**Results:**

This strain displays phenotypes that have been associated with chronic respiratory infections in CF including alginate over-production, rough lipopolysaccharide, quorum-sensing deficiency, loss of motility, decreased protease secretion, and hypermutation. Hypermutation is a key adaptation of this bacterium during the course of chronic respiratory infections and analysis indicates that PAHM4 encodes a mutated *mutS* gene responsible for a ~1,000-fold increase in mutation rate compared to wild-type laboratory strain *P. aeruginosa* PAO1. Antibiotic resistance profiles and sequence data indicate that this strain acquired numerous mutations associated with increased resistance levels to β-lactams, aminoglycosides, and fluoroquinolones when compared to PAO1. Sequencing of PAHM4 revealed a 6.38 Mbp genome, 5.9 % of which were unrecognized in previously reported *P. aeruginosa* genome sequences. Transcriptome analysis suggests a general down-regulation of virulence factors, while metabolism of amino acids and lipids is up-regulated when compared to PAO1 and metabolic modeling identified further potential differences between PAO1 and PAHM4.

**Conclusions:**

This work provides insights into the potential differential adaptation of this bacterium to the lung of patients with bronchiectasis compared to other clinical settings such as cystic fibrosis, findings that should aid the development of disease-appropriate treatment strategies for *P. aeruginosa* infections.

**Electronic supplementary material:**

The online version of this article (doi:10.1186/s12864-015-2069-0) contains supplementary material, which is available to authorized users.

## Background

*Pseudomonas aeruginosa* is an adaptable and resilient Gram-negative bacterium found ubiquitously in the environment [1–3] that is capable of infecting a wide range of organisms, including vertebrates, invertebrate eukaryotes, and plants [4]. In humans, it is responsible for causing keratitis, burn wound infections, and severe chronic respiratory infections in patients with underlying diseases such as cystic fibrosis (CF) or bronchiectasis [5]. The ubiquitous nature of *P. aeruginosa* and range of diseases is likely due in part to high genome plasticity [6].

Bronchiectasis is a pulmonary disease characterized by dilated bronchi, airway inflammation, chronic sputum production, and long-term bacterial colonization resulting in frequent exacerbations of bacterial infections [7]. Chronic *P. aeruginosa* infections in these patients are often associated with a worsening of symptoms, decreased pulmonary function, and increased frequency of exacerbations [8]. Once established, these infections are difficult to treat with antibiotics. The chronic lung inflammation, airflow obstruction, and extensive tissue remodeling found in the lungs of bronchiectasis patients resemble those of patients with CF or chronic obstructive pulmonary disease (COPD) [9]. The adaptation of *P. aeruginosa* to the CF lung has been extensively studied during the past decade [10–16]; however, little is known about the molecular mechanisms underlying the persistence of this bacterium in the lungs of patients with bronchiectasis.

A model describing the increased fitness in a chronic infection has been previously established (reviewed by Montanari et al. [17]) and this process can be enhanced by mutations to the various DNA proofreading systems, result in an increased mutation rate. During chronic CF infections, *P. aeruginosa* “hypermutators” can be isolated from 37 to 54 % of the patients [18, 19]. MutS, a critical component of the mismatch repair system [20], is commonly lost in hypermutator strains [19], resulting in elevated mutation rates [21]. *P. aeruginosa* hypermutator strains isolated from chronically infected patients, including those with bronchiectasis [22], are often more resistant to antibiotics, possess a mucoid phenotype, as well as loss of lipopolysaccharide (LPS) O-antigen and motility [15].

In this work, we have determined the genome sequence and characterized numerous phenotypes of *P. aeruginosa* strain PAHM4, a hypermutator from a chronic bronchiectasis infection that has been the subject of previous research [23]. This strain has several unique DNA islands and characteristics that may have facilitated the persistence of the microorganism in the lung. While this strain shares some traits with chronic CF isolates, it also differs from CF isolates in numerous ways, potentially highlighting differences between the CF lung and the bronchiectasis lung. By identifying key characteristics required for specific lung infections such as the expression of specific virulence factors, antigens or antibiotic resistance genes, it may be possible to identify drug targets specific to each type of infection. These findings have the potential to aid in the development of infection-specific treatments for *P. aeruginosa*.

## Results and discussion

### Virulence phenotypes of *P. aeruginosa* PAHM4 differ from those of CF and laboratory isolates

*P. aeruginosa* PAHM4 has been previously studied due to its constitutive trimethylation of EF-Tu, which mimics platelet activating factor and binds to platelet activating factor receptor [23, 24]. During these investigations, other interesting phenotypes of this strain, such as mucoidy and hypermutation, were observed when compared to chronic CF or laboratory isolates prompting an in depth characterization of the strain.

**Fig. 1:**
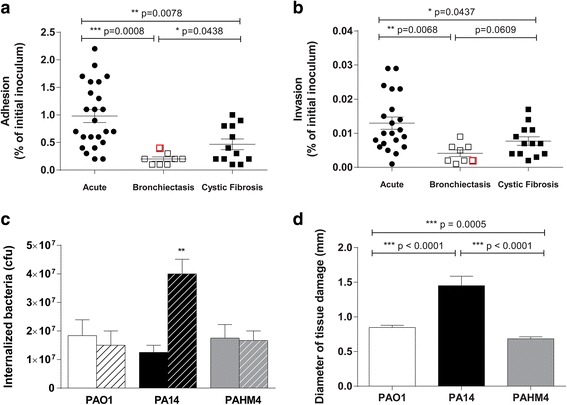
*In vitro* models of infection. Adhesion (**a**) and invasion (**b**) assays of individual clinical isolates from patients with acute pulmonary infections, bronchiectasis, or CF. Type II pneumocytes A549 cells monolayers were infected with 5 x10^5^ CFU and the percentage of adhered and invaded cells was measured using a standard gentamycin exclusion assay. PAHM4 is highlighted on the graphs in red. Experiments were performed in triplicate. C. Macrophage uptake (solid bars, time = 15 min) and killing (striped bars, time = 90 min) were performed using murine bone marrow macrophages extracted from BALB/c mice and grown in presence of L-cells conditioned medium for 5 days. Macrophages were infected with PAO1, PA14 or PAHM4 at a MOI 500:1. Experiments were performed in duplicate. D. *In vitro* infection of lettuce leaves. The midrib of romaine lettuce was infected with a bacterial suspension of 10^6^ CFU of PAO1, PA14 or PAHM4 and incubated at ambient temperature for 48 h. The diameter of the soft rot area at the site of inoculation was measured. Experiments were performed in triplicate. For all experiments, data were analyzed using an unpaired two-tailed *t*-test and the software GraphPad Prism 5.01

Adhesion and invasion assays indicated that PAHM4 and other bronchiectasis isolates differed from acute lung infection and chronic CF isolates. Strains isolated from acute infections displayed a significantly higher adhesion (Fig. 1a) and invasion (Fig. 1b) capacity compared to strains isolated from patients with bronchiectasis (*p* = 0.0008 and *p* = 0.0068, respectively, unpaired *t*-test) or cystic fibrosis (*p* = 0.0078 and *p* = 0.0437, respectively, unpaired *t*-test). PAHM4 adhesion and invasion capacity was low, likely due to its hyper-mucoid phenotype, but not significantly different from the other bronchiectasis isolates. Interestingly, we observed that overall adhesion and invasion of these bronchiectasis isolates was significantly lower (~0.43-fold and ~0.86-fold, respectively) than the isolates from CF infections, suggesting that adaptation of *P. aeruginosa* isolates to the lung of patients with bronchiectasis results in the selection of different phenotypes.

The capacity of PAHM4 to evade phagocytosis and to survive intracellularly in macrophages was also examined. Naïve bone marrow-derived macrophages from BALB/c mice were infected with 2.5 x 10^8^ CFU/well (MOI 500:1) of *P. aeruginosa*. Intracellular bacteria were quantified after 15 min incubation to measure uptake, or a 90 min incubation to measure intracellular survival. No significant difference in macrophage uptake was observed between the different strains tested (Fig. 1c, solid bars). After 90 min incubation, the number of live intracellular PAO1 and PAHM4 did not significantly change (*p* = 0.6703 and *p* = 0.9001, respectively) compared to the number of intracellular bacteria after 15 min, whereas the number of live PA14 had tripled (*p* = 0.0036). Intracellular PAHM4 populations were maintained during the length of the experiments, indicating that PAHM4 is resistant to murine macrophage killing in this experimental setting similar to PAO1, but in contrast to the PA14 population that increased during the course of the experiment (Fig. 1c, striped bars).

Lettuce leaves were infected with PAO1 (burn), PA14 (wound) and PAHM4 (non-CF bronchiectasis) to investigate the virulence of these *P. aeruginosa* strains in an environmental setting. In this model, PAHM4 displayed a significantly decreased capacity of infection compared to PAO1 and PA14 (~0.8-fold, *p* < 0.0005, Fig. 1d).

**Fig. 2:**
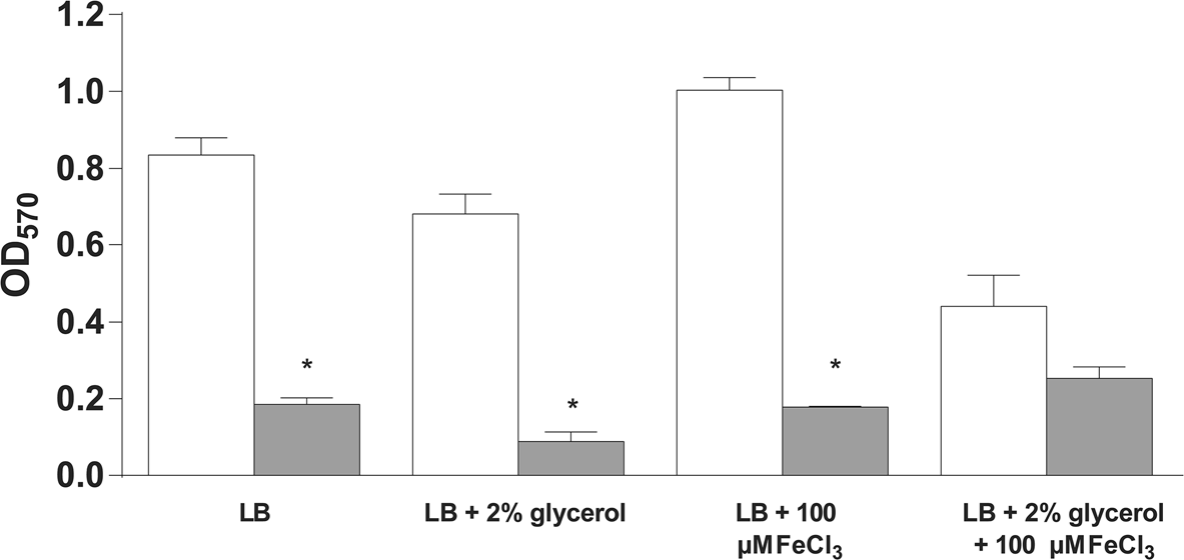
Biofilm formation in PAHM4. Measurement of biofilm formation of PAO1 (white bars) and PAHM4 (grey bars) grown for 24 h in rich media (LB) either unsupplemented or with glycerol, iron, or iron and glycerol added. *, *p* < 0.05. Error bars represent the standard deviation of biological and technical replicates

Biofilm production is thought to be a hallmark of chronic colonization of the CF lung [25], having been indirectly [26, 27] and directly [28] observed in CF sputum and lungs. Accordingly, the capacity of PAHM4 to form biofilms in rich media (LB), with and without supplementation with iron and/or glycerol, was determined and compared to PAO1. PAO1 formed significantly more biofilm than PAHM4 in lysogeny broth (LB) (*p* = 0.0154), in the presence of glycerol (*p* = 0.0161) and iron (*p* = 0.0177) (Fig. 2). It is important to note that while PAHM4 exhibited slower growth than PAO1 in LB with shaking at 37 °C (data not shown), it has been reported that growth rate does not necessarily influence biofilm formation [29]. Biofilm formation can be variable in individual CF isolates, even well studied strains like LESB58, which has variously been reported as hyper- [30], hypo- [31], or an equivalent- [32] producer of biofilms compared to PAO1.

**Fig. 3:**
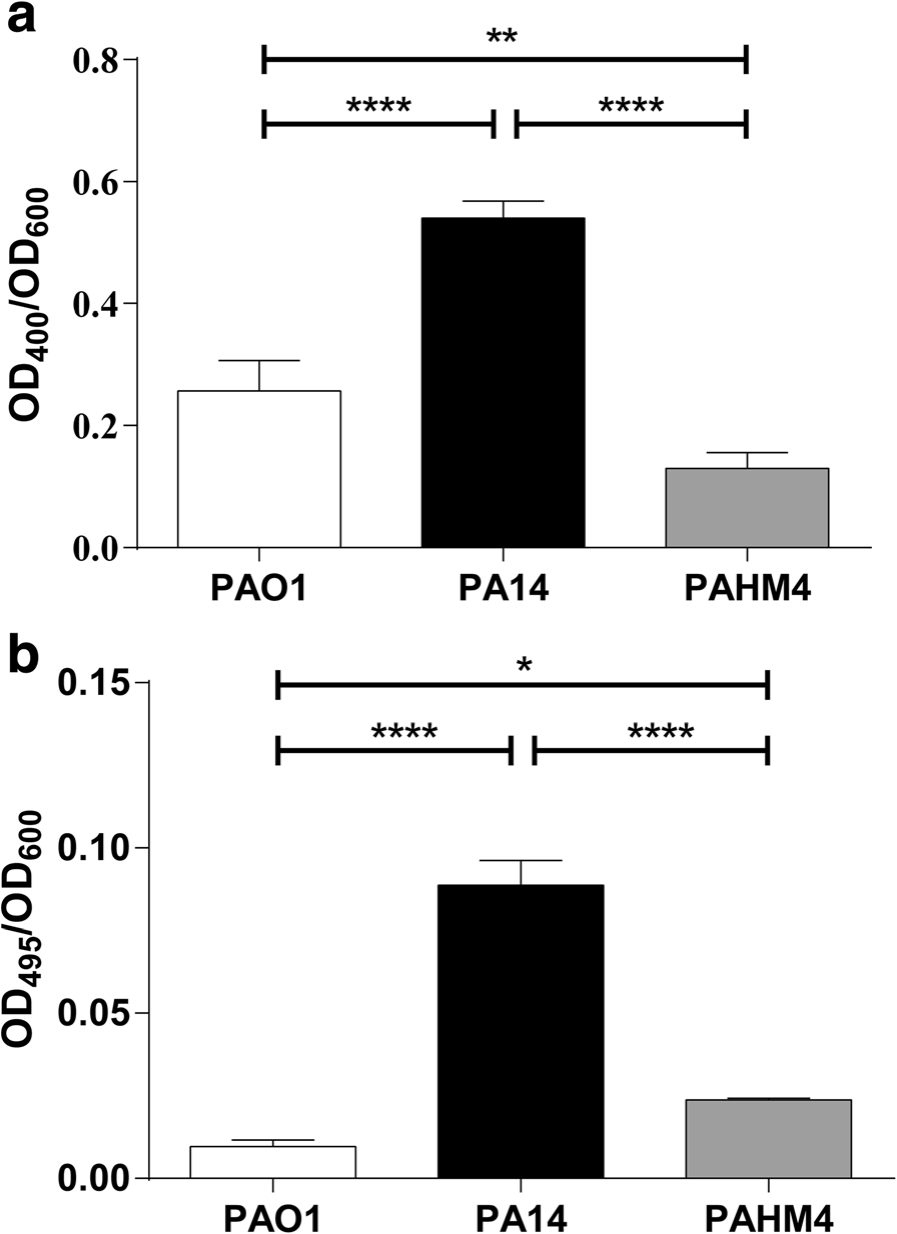
Protease and elastase assays. **a** PAHM4, PAO1, and PA14 were grown overnight in LB and filtered culture supernatants were added to an azocasein solution. After incubation, trichloroacetic acid was added to precipitate the remaining proteins and the OD_495_ was measured. Data presented are the OD_495_/OD_600_ of the overnight culture. **b** Elastase assays were performed by adding the filtered culture supernatant to an elastin-congo red solution and measuring the OD_400_ after 2 h. All assays were performed in triplicate, and the mean is plotted with error bars representing the standard deviation. Based on Tukey’s multiple comparison test: *, *p* = 0.0217,**, *p* = 0.0085, ****, *p* = < 0.0001

*P. aeruginosa* secretes numerous enzymatic virulence factors, including proteases, elastase, and hemolysins. *P. aeruginosa* proteolysis is due to secretion of a type IV protease, elastase, and an alkaline protease. Quantitative casein degradation assays indicated that PAHM4 had significantly less general protease activity than PA14 and PAO1 (Fig. 3a). The elastase LasB is involved in the disruption of tight junctions, and the cleavage of numerous proteins including surfactants, cytokines, chemokines, C3, and immunoglobulin [33, 34]. Elastase activity, as measured by the degradation of elastin-congo red elastin, was significantly decreased in PAHM4 compared to PA14 and significantly more than PAO1 (Fig. 3b). While protease and elastase levels vary between PAO1 and PA14, PAHM4 seems to have reduced overall protease levels while retaining elevated elastase activity compared to PAO1. This contrasts typical mucoid CF isolates that routinely lose elastase activity [35–37] in addition to having reduced levels of general protease activity [38].

*P. aeruginosa* hemolysis is due to two hemolytic extracellular products: phospholipase C, a heat-labile hemolysin, and a rhamnolipid, a heat-stable hemolysin. Assessment of the hemolytic capacity of PAHM4 after growth for 24 h on blood-agar plates indicates the presence of β-hemolysis activity in PAHM4 [39] (data not shown).

### PAHM4 phenotypes in common with CF isolates

Despite these differences when compared to *P. aeruginosa* laboratory strains and previously reported for CF isolates, PAHM4 and CF isolates have several phenotypes in common including mucoidy, hyper-mutation, loss of motility, and an extensive antibiotic resistance profile.

PAHM4 colonies appeared to be mucoid under typical growth conditions, similar to chronic CF isolates. Quantification of alginate indicates that PAHM4 produces 10 times more alginate that PAO1 in the tested conditions, with an average of 11.01 μg / 10^9^ cfu (*p* < 0.0001 unpaired *t*-test).

To characterize the hypermutator phenotype in PAHM4, the frequency of emergence of rifampicin-resistance was used to calculate the mutation rate (Table 1). Wild-type PAO1 had a mutant frequency of 4.9 x 10^−9^ and a mutation rate of 1.8 x 10^−9^ mutations/cell/generation, while a Δ*mutS* isogenic strain, PAOMS, showed a 2,000-fold increase in mutation rate (Table 1). The mutant frequency and mutation rate of PAHM4 were equivalent to the PAO1 Δ*mutS* strain, and complementation of PAHM4 with *mutS* from PAO1 reduced the mutant frequency and mutation rate by nearly 2 logs (Table 1), leaving a residual 10-fold increase in mutation rate. This indicates that the *mutS* mutation is the major cause of the hypermutation phenotype observed in PAHM4, similar to what is seen in CF isolates [40].

**Table 1:** *mutS*-related mutant frequency and mutation rate in PAHM4

Strain	Mutant frequency	Mutation rate (±95 % CI)
PAO1	4.9 x 10^−9^	1.8 x 10^−9^ (1.2–2.7)
PAOMS^a^	2.5 x 10^−6^	3.8 x 10^−7^ (2.4–4.2)
PAHM4	2.2 x 10^−6^	1.9 x 10^−7^ (1.5–2.4)
PAHM4 (pUCPMS)^b^	7.0 x 10^−8^	2.3 x 10^−8^ (1.6–3.1)
PAHM4 (pUCPML)^c^	2.5 x 10^−6^	3.3 x 10^−7^ (2.7–4.1)
PAHM4 (pLM102)^d^	1.5 x 10^−6^	1.9 x 10^−7^ (1.5–2.3)

^a^ *mutS* mutant

^b^cloned PAO1 *mutS*

^c^cloned PAO1 *mutL*

^d^empty vector

It is possible that the remaining increase in mutation rate in the *mutS* complemented strain was due mutations in *mutL* or *mutY*. To test this hypothesis, plasmids encoding wild-type *mutL* and *mutY* were used to measure the change in mutation frequency. In contrast to *mutS*, complementation with wild-type *mutL* or *mutY* had no effect on mutant frequency and mutation rate, suggesting a marginal impact, if any, of mutations to these genes in PAHM4.

**Fig. 4:**
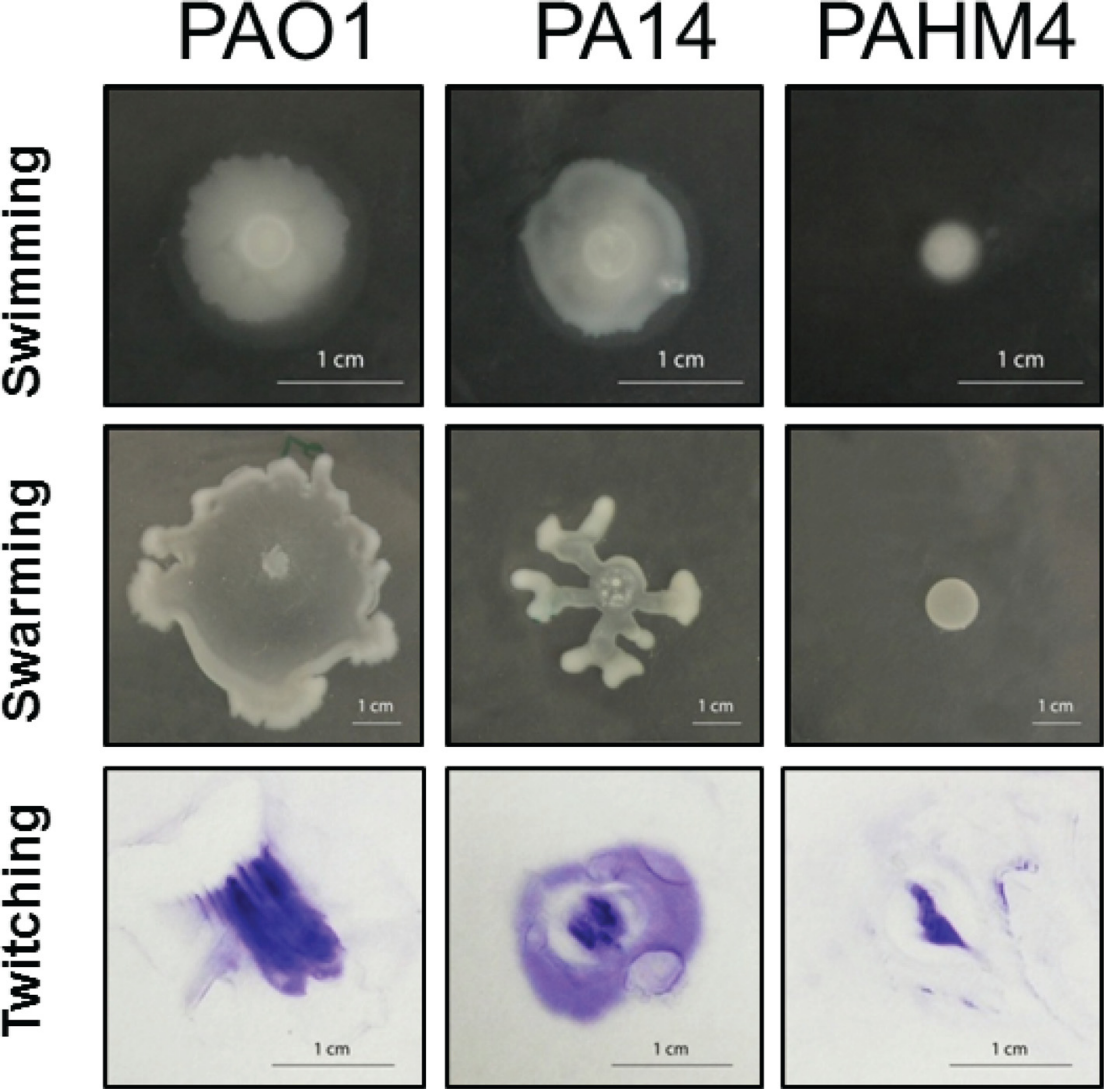
Phenotypic analysis of PAHM4. Visualization of swimming, swarming and twitching motility in media containing 0.3 % agarose, 0.5 % agar and 1.2 % agar, respectively. Motility was assessed after 48 h at 37 °C and experiments were performed in triplicate

The loss of both flagellar- and type IV pilus (TFP)- mediated motility is often observed in chronic CF isolates [41, 42]. A phenotypic switch to non-motility has also been noted in COPD isolates [43]. Compared to PAO1 and PA14, PAHM4 was defective for flagellar motility on semisolid media (0.3 % agar for swimming and 0.5 % agar for swarming) and twitching motility (1 % agar) (Fig. 4). These results are similar to those seen in LESB58, which has been reported to be defective in swimming and swarming motility [30, 31], while totally lacking twitching motility [31].

**Fig. 5:**
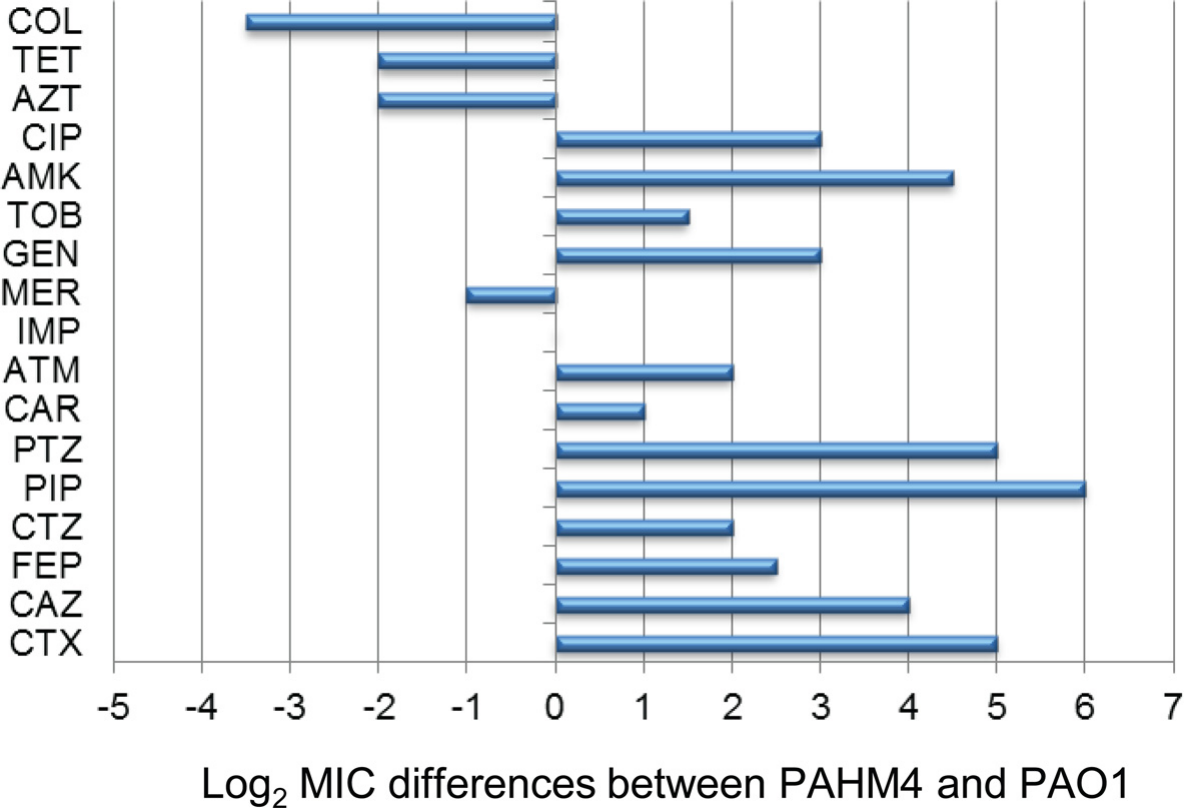
Antibiotic susceptibility profile. Comparative analysis of the antibiotic susceptibility profile of strain PAHM4 with that of PAO1 with values reported as the Log_2_ of MIC differences (positive values are increased resistance, negative values are decreased resistance of PAHM4 compared to PAO1). Antibiotics tested are cefotaxime (CTX), ceftazidime (CAZ), cefepime (FEP), piperacillin (PIP), piperacillin-tazobactam (PTZ), aztreonam (ATM), imipenem (IMP), meropenem (MER), ciprofloxacin (CIP), gentamicin (GEN), tobramycin (TOB), amikacin (AMK) and colistin (COL) tetracycline (TET), azithromycin (AZT), ceftolozane (CTZ) and carbenicillin (CAR)

Patients with a chronic respiratory infection are subject to heavy antibiotic treatment. The survival of *P. aeruginosa* in the chronically infected lung is often associated with an increased resistance to antimicrobial agents. PAHM4 is clinically resistant to penicillins (carbenicillin, piperacillin and piperacillin in combination with tazobactam) and aminoglycosides (amikacin and gentamycin) (Table 2 and Fig. 5). This strain also displays significantly higher levels of resistance to cephalosporins, including cefotaxime and monobactams, and fluoroquinolones, when compared to PAO1. However, PAHM4 has increased susceptibility to carbapenems (meropenem), polymyxins, macrolides and tetracycline compared to PAO1 (Table 2 and Fig. 5). Intermediate resistance levels to ceftazidime and ciprofloxacin in PAHM4 are most likely associated with the prolonged use of these antibiotics for the treatment of this patient.

**Table 2:** MIC values for common clinical antibiotics against PAHM4 and PAO1

Class	Antibiotic	MIC μg/ml for PAO1	MIC μg/ml for HM4
Penicillins	Piperacillin	4 (S)	>256 (R)
	Piperacillin-Tazobactam	2 (S)	>256 (R)
	Carbenicillin	32	64
Cephalosporins	Ceftazidime	1 (S)	16 (I)
	Cefepime	1 (S)	6 (S)
	Cefotaxime	8	>256
	Ceftolozane	0.25	1
Monobactam	Aztreonam	2 (S)	8 (S)
Carbapenems	Imipenem	1.5 (S)	1.5 (S)
	Meropenem	0.38 (S)	0.19 (S)
Quinolones	Ciprofloxacin	0.125 (S)	2 (I)
Aminoglycoside	Amikacin	4 (S)	96 (R)
	Gentamicin	2 (S)	32 (R)
	Tobramycin	1.5 (S)	4 (S)
Polymyxin	Colistin	2 (S)	0.19 (S)
Tetracycline	Tetracycline	16	4
Macrolide	Azithromycin	64	16

Absolute MIC values and, when available, *CLSI* clinical categories (S: susceptible, I: intermediate, R: resistant) are indicated

Penicillin, cephalosporin and monobactam resistance is most likely due to the activity of the inducible β-lactamase AmpC encoded by *ampC* on the chromosome of *P. aeruginosa*. High resistance levels to these β-lactams are often associated in *P. aeruginosa* clinical isolates with mutations in the genes *ampD* and *dacB*, encoding AmpD, a repressor of *ampC* expression, and the penicillin binding protein 4, respectively. In other strains, mutations in *ampD* and *dacB* result in the derepression of *ampC* expression [44]. Mutation of *dacB* additionally derepresses the expression of *creD* through the activation of the CreBC two-component regulator, contributing further to β-lactams resistance [45]. To address these considerations, a series of reverse transcription-quantitative PCR (RT-qPCR) assays were performed to probe the nature of antibiotic resistance in PAHM4. Our data indicate that *ampC* and *creD* basal expression in PAHM4 are significantly higher than in PAO1 (Table 3), and that *ampC* expression in PAHM4 is not inducible. These results indicate that the expression of *ampC* is fully derepressed, a phenomenon often observed in Gram-negative bacteria isolated from patients undergoing therapy with third-generation cephalosporins, such as cefotaxime and ceftazidime [46, 47].

**Table 3:** RT-qPCR expression levels of antibiotic determinants

	PAO1		HM4	
	Basal Expression	Induced expression^a^	Basal Expression	Induced expression^a^
*ampC*	1	329 ± 8	509 ± 4	512 ± 181
*creD*	1	32 ± 12	32 ± 13	25 ± 7
*mexB*	1		0.006 ± 0.002	
*mexD*	1		0.007 ± 0.001	
*mexF*	1		2.1 ± 0.2	
*mexY*	1		30 ± 11	

Mean values of relative (compared to PAO1) mRNA levels obtained in at least three independent duplicate experiments. ^a^For *ampC* and *creD* induction experiments, cultures were incubated in the presence of 50 μg/mL cefoxitin. Cefoxitin is an inducer of AmpC (and CreD) expression but does not affect efflux pump (*mexBDFY*) expression

### Genome sequence determination of PAHM4

Given the interesting mix of phenotypes observed in PAHM4, the genome sequence was determined, allowing for the identification of the genetic basis for the observed phenotypes, as well as potentially identifying additional unique features of a non-CF *P. aeruginosa* lung isolate. Sequence determination resulted in an apparent genome size of 6,381,186 bp encoding 5906 putative ORFs and 62 structural RNAs. The genome sequence and protein sequences used for all downstream analyses are included as Additional file 1: Data File 1 and Additional file 2: Data File 2, respectively. The PAHM4 genome has a size, coding density, and % G + C similar to other *P. aeruginosa* strains (Table 4). MLST analysis indicates that PAHM4 belongs to *P. aeruginosa* MLST clone ST-195 [48], which is comprised of 11 strains included in the MLST database (pubmlst.org/paeruginosa, last accessed 6/17/2014). Ten of eleven strains of this MLST type were isolated from CF patients in France or Australia, suggesting a propensity of this clonal lineage for developing chronic infections, while the other was a canine isolate.

**Table 4:** Genomic characteristics of select *P. aeruginosa* strains

Strain	PAHM4	PAO1	PA14	PA2192^c^	PA7	LESB58	NCGM2.S1	C3719^c^
Size (bp)	6,381,186	6,264,403	6,524,142	6,905,121	6,588,339	6,601,757	6,764,661	6,222,097
% G + C	66.2	66.6	66.3	66.2	66.5	66.5	66.1	66.5
# ORFs	5906	5686	5584	6191	6071	6113	6287	5578
Average ORF size	938	997	1022	949	954	958	956	943
Coding %	87.4	90.4	87.4	85.7	87.9	88.7	88.9	87.4
rRNA	12	13^b^	13^a,b^	4	12	12	12	4
tRNA	55	63	59^a^	46	65	68	66	40

^a^Values from pseudomonas.com [147]

^b^includes annotated 6S rRNA

^c^Values from Mathee *et al.* [169]

Genome plasticity and a large accessory genome are keys for adaptation of *P. aeruginosa* to a wide range of environments [6]. Circular genome comparisons generated with BRIG [49] that show large scale differences between the *P. aeruginosa* genomes are included as Additional file 3: Data File 5, using PAO1 as the reference, and Additional file 4: Data File 6, using a PAHM4 pseudochromosome as the reference. To understand the extent of genome plasticity in PAHM4, and the potential role it played in adaptation to the bronchiectasis lung, PanSeq [50] was used to compare the genome of PAHM4 to the 7 reference strains listed in Table 4.

These comparisons revealed that PAHM4 has at least 117 genome islands (GI), ranging in size from 399 – 49,917 bp, containing 377,121 bp of DNA not found in other analyzed *P. aeruginosa* strains. Nineteen of these GIs, totaling 33,157 bp, had no significant BLASTN hits (cutoff e = 0.01) in the non-redundant database as of 7/1/2014.

PAHM4 DNA regions that were not present in other *P. aeruginosa* strains were analyzed with BLASTX to identify potential proteins. Numerous regions of interest were identified, including prophage, a serotype O13 LPS O-antigen locus, several type 6 secretion system (T6SS) loci, putative virulence factors, metabolic genes, fimbriae and pili, and a putative steroid degradation cluster (Additional file 5: Table S1). This latter region consists of 6 ORFs that have high DNA and protein similarity to a steroid degradation gene cluster identified in *Pseudomonas resinovorans* (GenBank accession AB74080). As inhaled steroids have historically seen use as supportive treatment for bronchiectasis [51, 52] the presence of this cluster in PAHM4 is intriguing as it suggests that this bacteria may be able to utilize steroids as an energy source, which might in turn negate the effectiveness of this treatment.

PanSeq also identified commonly occurring DNA sequences from *P. aeruginosa* strains that were absent from the PAHM4 genome. This analysis identified 42 segments of DNA comprising 172,189 bp that were present in all seven other analyzed strains and missing in PAHM4 (Additional file 5: Table S2). The two largest clusters accounted for ~33 % of the total common DNA absent from PAHM4 (Additional file 5: Table S2). The first large deletion in the PAHM4 genome spans PAO1 ORFs PA1335 and PA1437 and includes the LasIR quorum sensing (QS) system (PA1431 and PA1432). The second large deletion includes PAO1 ORFs PA2128-PA2192. Of these, PA2128-PA2181 have previously been reported to be involved in biofilm formation [53] and under the control of the transcriptional regulator RpoN in a mucoid strain of *P. aeruginosa* [54]. These genes are also involved in carbohydrate metabolism, and their loss might be the result of the metabolic adaptation of PAHM4 to chronic infection, as described below.

Bacterial genomes often contain prophages or prophage-like elements which can differ between individual isolates of the same species and provide valuable information on bacterial evolution. Prophages mediate horizontal transfer of genetic material through transduction and provide important biological properties to their hosts. Large, apparently intact but not necessarily complete prophage sequences were identified using PHAST [55] while ProphageFinder [56] was used to more aggressively analyze *P. aeruginosa* genomes for potential prophage content in the form of degraded phage genomes. Similar to observations in other *P. aeruginosa* strains, PAHM4 had several common prophage as well as a complement of unique sequences (Additional file 5: Table S3).

### Genome analysis identified several phenotypes common to PAHM4 and CF isolates

Above, we have shown that hyper-mutation of PAHM4 is primarily the result of a deficiency in *mutS*. Sequence analysis of the *mutS* locus in *P. aeruginosa* PAHM4 revealed a 2,559 bp ORF which has 98 % identity to PAO1 *mutS* (PA3620). Compared to PAO1, *mutS* from PAHM4 has several SNPs and an 1184del9 mutation. Alignment of the resulting PAHM4 protein to the PAO1 protein identifies two changes: S281G and ΔT395_G397. Warren and colleagues identified a *P. aeruginosa* isolate from a chronic infection that also possessed the S281G mutation and additional isolates in which MutS was truncated at residue 426 [11]. BLASTP analysis indicates that the specific three residue deletion in PAHM4 is a unique occurrence among MutS from *P. aeruginosa* strains and other members of the genus [11, 57].

As described above, PAHM4 over-produces alginate. This phenotype is often associated with the establishment of chronic respiratory infections by *P. aeruginosa*. PAHM4 *mucA* contains a ΔG430 mutation, causing a premature stop codon and resulting in a mutant allele commonly referred to as *mucA22* [58, 59]. *mucA22* strains are constitutively mucoid, and this is the apparent cause of alginate overproduction by PAHM4 [60]. In the lungs of patients with CF, 85 % of mucoid *P. aeruginosa* isolates have mutations in *mucA* [61], with *mucA22* being the most common allele [62].

In addition to alginate, *P. aeruginosa* strains produce other exo-polysaccharides known as Pel and Psl [63]. PAHM4 encodes an intact Pel locus while the Psl locus has an assembly gap within *pslL*.

**Fig. 6:**
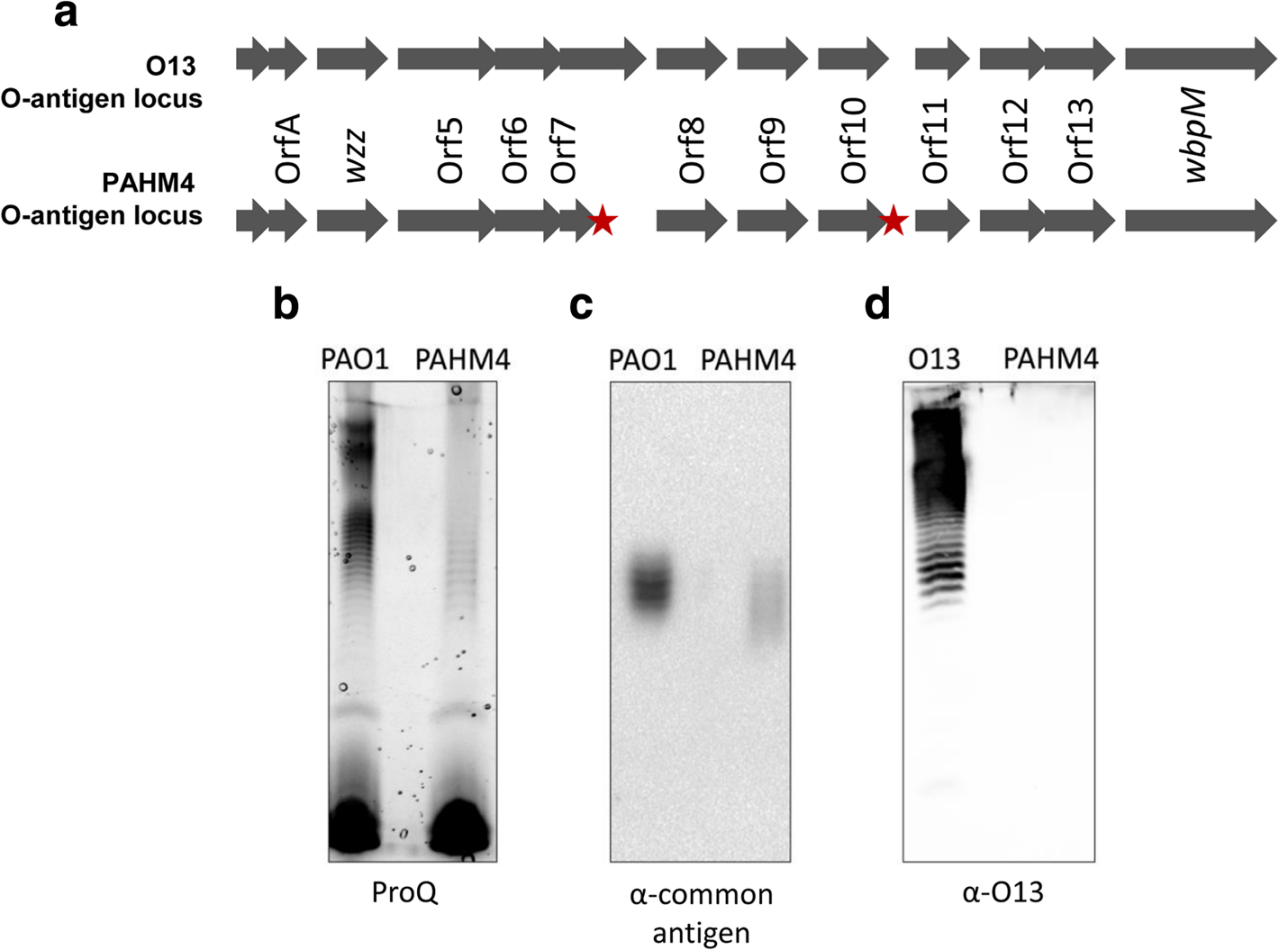
O-antigen biosynthetic locus and LPS expression in PAHM4. **a** Genetic comparison of LPS O-antigen biosynthetic regions of serotype O13 (top) and PAHM4 (bottom). Gene names are as described by Raymond *et al*. [66]. Stars denote the location of insertions in PAHM4 relative to the Serotype O13 reference sequence. **b** ProQ Emerald 300 lipopolysaccharide gel stain of PAO1 and PAHM4 LPS preparations. **c** Western blot of PAO1 and PAHM4 LPS preparations, probed with anti-common antigen antibodies. **d** Western blot of Serotype O13 and PAHM4 LPS preparations, probed with anti-O13 antibodies

*P. aeruginosa* strains produce a variety of serotype-specific O-antigen side chains on lipopolysaccharide (LPS) (reviewed by Knirel et al. [64]). The O-antigen are used as the basis for a typing scheme based on antibody agglutination [65] and each serotype has a corresponding genotype [66]. BLAST analysis of the PAHM4 genome using the O-antigen locus sequences from Raymond et al. [66] identified a 13,163 bp region in PAHM4 with 99.19 % identity to serotype O13 (Fig. 6a). The PAHM4 locus possesses two 1-bp insertions compared to serotype O13. The first disrupts ORF7, introducing several premature stop codons while the second is intergenic and does not appear to influence any genes (Fig. 6a). Strains corresponding to several serotypes (e.g. O1, O5, O6, O10) have been sequenced, however no serotype O13 strain has had its genome sequence determined and published. Chronic CF isolates are known to frequently lose the O-antigen from LPS, which is proposed to aid in immune evasion [10, 67–69]. ProQ staining of LPS preparations from PAO1 and PAHM4 revealed faint LPS banding (Fig. 6b). It was anticipated that this might be the common antigen portion of LPS, and a Western blot using anti-common antigen Mab demonstrated the presence of common antigen in PAHM4 (Fig. 6c). As expected given the mutation in ORF7, western blotting of the LPS preps using O13 antibodies did not indicate the presence of any O13 antigen (Fig. 6d).

**Fig. 7:**
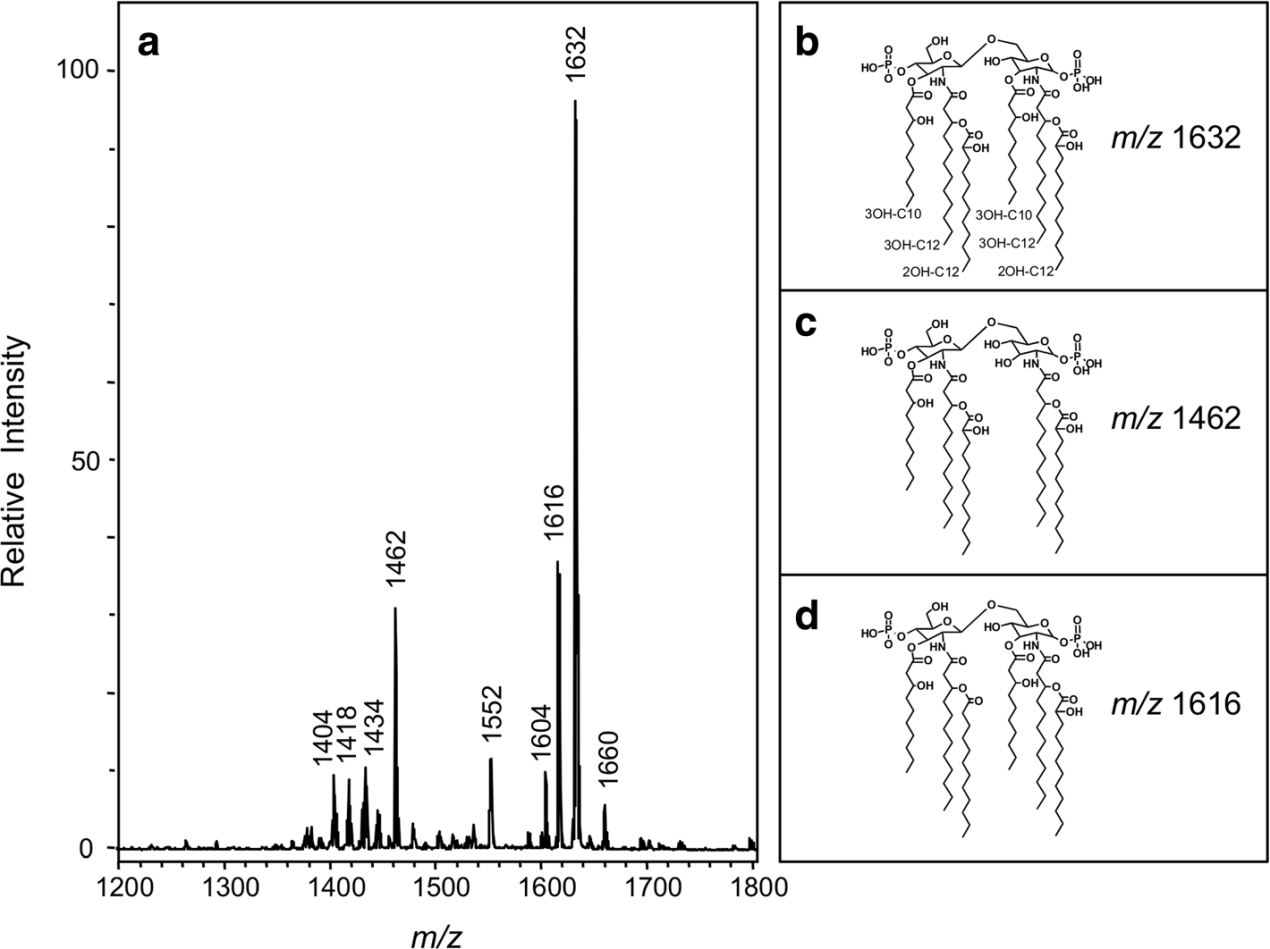
Lipid A analysis of PAHM4. MS analysis (panel **a**) revealed major lipid A species present at *m/z* 1632 (panel **b**) with minor species *m/z* 1462 (panel **b**) and *m/z* 1616 (panel **c**). Other ions present represent monophosphoryl species (*m/z* 1552), previously identified penta-acylated lipid A (*m/z* 1418), as well as non-hydroxylated lipid A (*m/z* 1600 and *m/z* 1432). Ions *m/z* 1404 and *m/z* 1660 have not been previously identified

Lipid A produced by PAHM4 after growth at 37 °C in LB with 1 mm MgCl_2_ was purified for structural analysis by MALDI-TOF MS in the negative ion mode. Results indicated that PAHM4 displayed lipid A species characteristic of *P. aeruginosa* (Fig. 7a). The most abundant anion giving the base peak at *m/z* 1632 corresponded to a singly deprotonated hexa-acylated lipid A (Fig. 7b). Specifically, PAMH4 synthesizes a hexa-acylated lipid A species containing hydroxylated fatty acids (2OH C12) attached acyl-oxo-acyl at the 2 and 2’ positions, as well as the retention of the 3OH-C10 fatty acid at the 3 position. The minor anion at *m/z* 1462 (penta-acylated) represents the loss of the 3 position 3OH C10 fatty acid from the *m/z* 1632 peak (1632–170 = 1462) (Fig. 7c), whereas the *m/z* 1616 (hexa-acylated) represents a structure that is only modified by a single hydroxyl group (1632 – 16 = 1616) (Fig. 7d). As compared to the structure of lipid A isolated from the laboratory adapted strains, PAK and PAO1, the lipid A produced by PAHM4 showed increased hydroxylation (OH group) and acylation. When compared to lipid A from a typical CF strain PAHM4 has a different hydroxylation pattern and lacks the addition of aminoarabinose [70]. The increased susceptibility of PAHM4 to polymyxins (Table 2) may be explained by the loss of the specific O-antigen, as suggested by earlier *Salmonella* studies [71] or by the alterations to lipid A [72].

### Genetic basis for antibiotic resistance

As described earlier, PAHM4 has resistance to many antibiotics, and trends in expression levels of resistance genes were similar to what is seen in chronic CF isolates. The genome sequence allows for identification of the basis for the observed resistances.

The high level of *ampC* and *creD* over-expression are consistent with PAHM4 sequence data showing changes present in AmpD (A85G, G148A and E170C) and DacB (A394P and G337D) compared to PAO1. Previous work showed that inactivation of these genes leads to a complete derepression of *ampC* expression [45]. *mexB* has a ΔC600 mutation, with the resulting frameshift causing a premature stop codon. These results are consistent with the meropenem hyper-susceptibility of this strain and, compared to PAO1, the relatively low increase in minimum inhibitory concentrations (MICs) for several of the β-lactams (particularly carbenicillin) despite the high level of *ampC* expression. Altogether, low levels of *mexB* expression and the mutation observed in this gene, which are presumably responsible for a non-functional protein, might account for the lower MICs of tetracycline and azithromycin of this strain. The sequences of *gyrA*, *gyrB*, *parC* and *parE* were analyzed in order to determine the source of fluoroquinolone resistance. Analysis identified a point mutation resulting in GyrA having a D87Y substitution that resides within the quinolone resistance determining region of the protein. This mutation, related to high levels of fluoroquinolone resistance, frequently occurs in CF isolates [73]. Other point mutations were observed in genes encoding ParC (S331T) and ParE (D533E), the latter being often associated with the GyrA D87Y substitution in *P. aeruginosa* clinical isolates [74], and possibly associated with the high quinolone resistance levels of the strain. The *gyrB* sequence in PAHM4 is identical to the PAO1 sequence. The selection of mutations in *gyrA*, *parC*, and *parA*, is likely the result of the use of ciprofloxacin for the treatment of the patient.

### PAHM4 has a deficiency for acyl homoserine lactone (AHL)-based quorum sensing

QS in *P. aeruginosa* is a well characterized phenomenon (reviewed by Williams and Camara [9]) that controls the expression of approximately 10 % of the genes in *P. aeruginosa* and is known to control virulence factor expression in *P. aeruginosa* as well as other microbial pathogens [75–77]. It has previously been reported that *P. aeruginosa* hypermutator strains frequently developed mutations in the *lasIR* QS system [78].

**Fig. 8:**
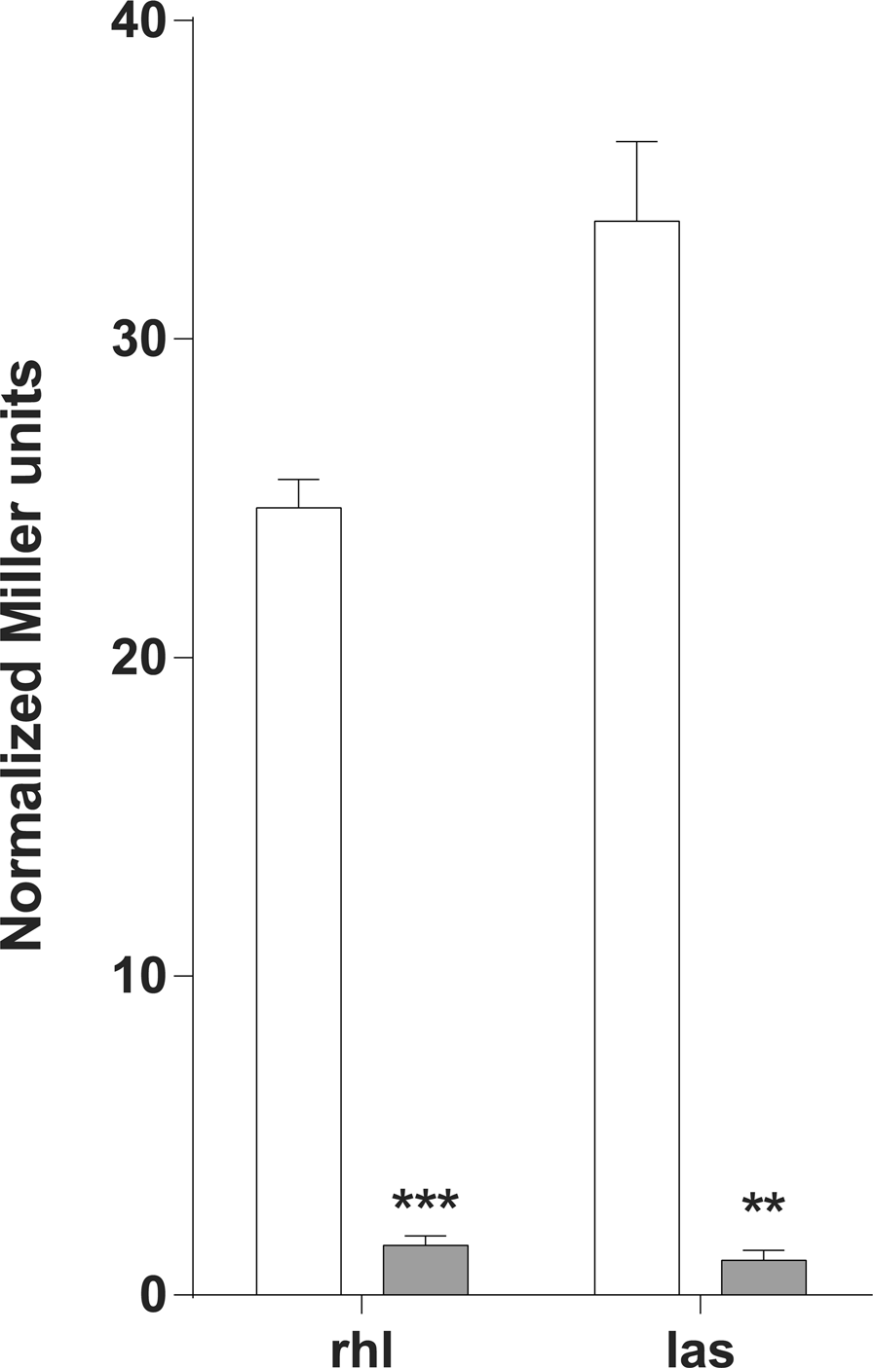
Presence of AHLs in PAHM4 culture supernatant. Measurement of the production of C4-acyl homoserine lactone and C12-acyl homoserine lactone using the PAO1 *rhl* reporter pEC65.1 and *las* reporters pJN105 and pSC11, respectively in PAO1 (white bars) and PAHM4 (grey bars)

As described above, sequence analysis indicated that a large chromosomal deletion in PAHM4 includes *lasI* and *lasR*. The *rhlIR* system is present and the resulting proteins are 99 % and 100 % identical, respectively, to the homologs in PAO1. Culture supernatants of late stationary phase PAHM4 and PAO1 samples were tested for the production of both *P. aeruginosa* autoinducers, C4-AHL (*rhlI*) and 3-oxo-C12-AHL (*lasI*) using *rhlR* and *lasR* based quorum sensing-responsive reporters [79, 80]. As expected, PAO1 culture supernatants activated both reporters, indicating the production of C4-AHL and 3-oxo-C12-AHL (Fig. 8). PAHM4 supernatant did not activate either reporter (Fig. 8), indicating an absence of both autoinducers; this was a surprising result given the intact state of the *rhlIR* genes. The CF isolate LESB58 belongs to a lineage known for increased C4-AHL production leading to over-production of QS-linked virulence factors and hyper-virulence [81]. However, the contrasting observations in PAHM4 are in line with findings from the longitudinal analysis of chronic CF isolates which indicated that typically *P. aeruginosa* strains would first lose Las activity while eventually also losing Rhl activity [82]. One mechanism for this is that the *mucA*22 allele, which is also present in PAHM4, has been shown to be required for full expression of *rhlIR* and *lasIR* [83]. Additionally, it is quite possible that the loss of detectable C4-AHL is influenced by base changes elsewhere on the chromosome.

### PAHM4 has an expanded arsenal of protein secretion apparatuses compared to other *P. aeruginosa* strains

Bacterial secretion systems play a major role in the export of proteins involved in virulence, nutrient scavenging, and immune evasion. The genome of PAHM4 was screened for the presence of *P. aeruginosa* secretion systems in order to understand the role of these systems in the adaptation of this strain to the chronic bronchiectasis lung.

**Fig. 9:**
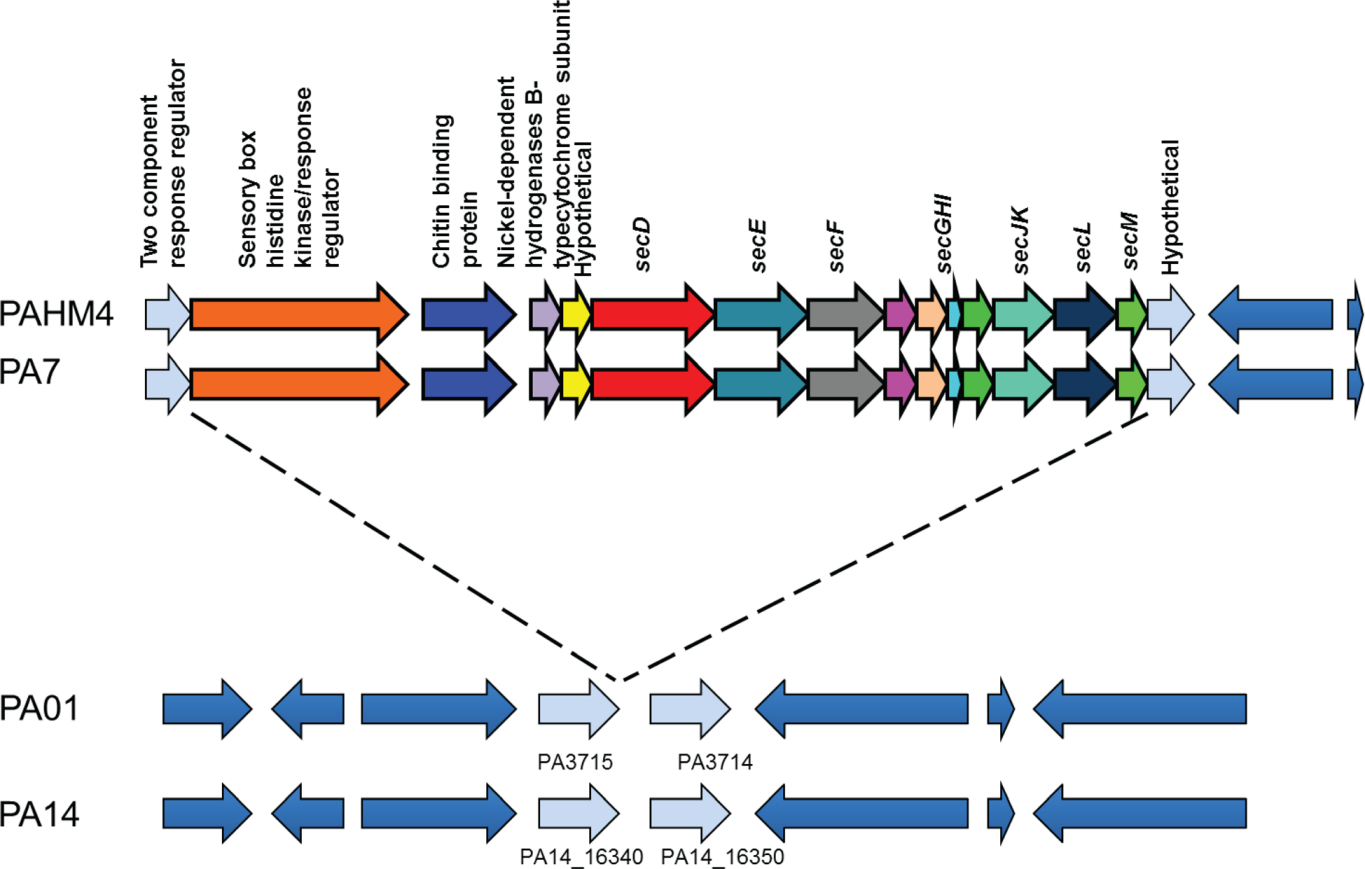
T2SS in PAHM4 and PA7. PAHM4 and PA7 encode a novel T2SS cluster that appears to have inserted between a two- component response regulator (PA3175) and a hypothetical protein (PA3714). The gene pairing of PA3714 and PA3715 homologs is maintained in other *P. aeruginosa* strains and sequences homologous to this T2SS are only found in *Culex pipiens quinquefasciatus*

PAHM4 contains four major operons encoding two distinct type 2 secretion systems (T2SS) that are present in the majority of *P. aeruginosa* strains. PAHM4 also contains a putative operon encoding a 3^rd^ T2SS (Fig. 9). This gene cluster shows ~90 % identity to the recently characterized Txc T2SS that had previously only been identified in the genome of *P. aeruginosa* PA7 [84, 85]. Intriguingly, the NCBI non-redundant database only contains several high quality matches to this sequence (sequence identity >80 %, query coverage >50 %) with the closest match after PA7 belonging to the mosquito, *Culex pipiens quinquefasciatus* [86]. This may be the result of an ancient horizontal gene transfer event between an endosymbiotic bacterium and the mosquito [87].

Another protein secretion mechanism important to *P. aeruginosa* virulence is the T6SS, which is a relatively recently described mechanism of protein export that is present in a wide array of Gram-negative bacteria. The T6SS complex requires numerous proteins for assembly of the apparatus and effector export [88–90]. Like other *P. aeruginosa* strains, PAHM4 encodes the canonical T6SS loci Hcp1 secretion island-1 (HSI-1), HSI-2, and HSI-3 [91]. In PAHM4, HSI-1, HSI-2 and HIS-3 are encoded on three separate contigs with over 98 % DNA identity to PA14 (Table 5).

**Table 5:** T6SS present in PAHM4

T6SS	*P. aeruginosa* PA14	*P. aeruginosa* PAO1	PAHM4 location	PAHM4 coordinates
HSI-1	PA14_00875-01110	PA0074-0091	assembly049	95451-97461
			assembly050	1-23876
HSI-2	PA14_42880-43100	PA1656-PA1671	assembly015	172345-195150
			assembly001	108099-110744
HSI-3	PA14_33940-34150	PA2359-PA2374	assembly024	37353-59972
PAHM4-specific			assembly028	495580-513221

Protein predictions indicate the presence of an additional T6SS system in PAHM4 located in a region of DNA not previously identified in *P. aeruginosa* strains that has homology to the *P. putida* genome. The majority of proteins in this cluster have homology to the type 1.2 T6SS cluster from *P. putida* [92]. This cluster is evolutionarily related to HSI-2 from *P. aeruginosa* but has never been detected outside of *P. putida* [92], suggesting that PAHM4 obtained it in a horizontal gene transfer event. Interestingly, this cluster contains several additional ORFs not seen in *P. putida*, and the predicted proteins showed homology to *Pseudomonas extremaustralis*, a species isolated from thawed ice in Antarctica [93, 94], and numerous other marine bacterial species, indicating a potential reservoir for this T6SS system.

PAHM4 encodes a robust assortment of homologs of Hcp and VgrG, secreted factors and structural components of T6SS [95–97]. PAHM4 is missing several conserved *P. aeruginosa hcp* and *vgrG* genes, but encodes additional homologs not found in PAO1 or PA14 (Additional file 5: Table S4).

### PAHM4 has a mix of novel and common cellular appendage alleles

*P. aeruginosa* cells are equipped with several appendages known to affect motility, biofilms, and adherence. Given the various phenotypes identified in PAHM4, the genes encoding these systems were investigated.

Type IV pili (TFP) are bacterial filaments typically composed of repeating monomers (reviewed by Pelicic [98]), which are known to have multiple functions in Gram-negative and Gram-positive bacteria including motility [99, 100] and biofilm formation [101, 102]. In *P. aeruginosa* TFP have known roles in twitching motility [103], biofilm formation [104], disease pathogenesis [105], and phage uptake [106].

*P. aeruginosa* produces two types of TFP: type IVa (TFPa) and type IVb (TFPb) [107–109]. Additionally, it has been shown that there are at least five distinct pilin alleles for TFPa pilin, identified by the genes inserted between tRNA-Thr and *pilA* [110], known collectively as group I-V. PAHM4, like PAO1 [111], encodes the group II TFPa allele which lacks accessory genes downstream of *pilA* [110] (Table 6). Group II TFPa is the most common allele identified in non-CF clinical isolates while group I makes up the majority of CF isolates [110]. *P. aeruginosa* strains also produce numerous minor pilins, encoded in an island downstream of the TFPa cluster [111, 112]. The minor pilins in PAHM4, encoded by *fimU*, and *pilVWXE*, are nearly 100 % identical to the pilins encoded by a diverse set of strains including PAO1, PA7, C3719, and NGCM2.S1 (Table 6).

**Table 6:** Analysis of TFP clusters in PAHM4

	PAHM4	PAO1	PA14	PA7	LESB58	C3719	NGCM2.S1
IVa group	2	2	3	4	1	3^a^	2^b^
minor pilin cluster type^c^	"PAO1"	"PAO1"	"PA14"	"PAO1"	"PAO1"	"PA14"	"PAO1"
IVb PAPI-1	+	-	+	-	-	-	-
IVb FLP	+	+	+	+	+	+	+

^a^C3719 glycosylation gene has a frameshift

^b^NGCM2.S1 *pilA* sequence is present but not annotated

^c^Minor pilin cluster type refers to the sequence being similar to the *fimU* and *pilVWXE* alleles present in either PAO1 or PA14

The PAHM4 genome contains the prototypical TFPb cluster seen in PA14 that encodes a bundle-forming pilus [109]. When compared to PA14, the coding sequence for this cluster was 99 % identical and all genes were intact. PAHM4 also contains a second TFPb cluster associated with the PAPI-1 element found in a subset of *P. aeruginosa* strains, including PA14 [108]. PAHM4 assembly043 contains a region with 98 % identity to a region spanning *pilL2* to *pilM2* in PA14, with all ORFs intact. This TFPb pilus is known to be required for the transmission of PAPI-1 [108], indicating that PAHM4 may retain the ability to transmit its PAPI-1-like element.

In addition to TFP, another surface filament present on *P. aeruginosa* cells is the chaperone-usher-pathway (CUP) fimbriae. *P. aeruginosa* strains are known to encode multiple, conserved, CUP systems [113]. PAHM4 and PA7 are missing the CupA cluster and have distinct unique alleles for CupB and CupC (Table 7). The CupA cluster is located within one of the large chromosomal deletions in PAHM4, while in PA7 the deletion is small and primarily consists of the CupA cluster (data not shown). The PAPI-1-associated CupD is absent in PAHM4 while CupE is conserved. CupA, CupB and CupC have been shown to be required for biofilm formation [113, 114] while CupE was shown to be required more specifically for the early phases of biofilm formation [115]. It is intriguing that PAHM4 and PA7 introduce new alleles of CupB and CupC while every other analyzed *P. aeruginosa* strain had an identical or nearly identical set of alleles.

**Table 7:** CUP cluster composition of *P. aeruginosa* strains

CUP cluster	PAHM4	PA7	PA14	NCGM2.S1	PA2192	PAO1	LESB58	C3719	M18
A	absent	absent	present	present	present	present	present	present	present
B^a^	PAHM4	PA7	PAO1	PAO1	PAO1	PAO1	PAO1	PAO1	PAO1
C^a^	PAHM4	PA7	PAO1	PAO1	PAO1	PAO1	PAO1	PAO1	PAO1
D^b^	absent	PAPI-1	PAPI-1	absent	absent	absent	absent	absent	absent
E	present	present	present	present	present	present	present	present	present
Other cluster^a,c^	absent	absent	absent	Absent	PA2192	PAO1	PAO1	PAO1	PAO1

^a^strain name indicates presence of a strain-specific allele

^b^CUP cluster D is associated with PAPI-1 island

^c^The unnamed cluster is described by Filloux [170]

*P. aeruginosa* expresses a single polar flagellum which exhibits a conserved structure established for many Gram-negative microorganisms. This flagellum is involved in motility and pathogenesis, promoting the attachment of the bacterium to the surface of epithelial cells. Genetic analysis of PAHM4 indicates that most flagellar biosynthesis genes in this bacterium are highly conserved compared to PAO1 including the genes *flgBCDEFGHIJKL*, *fleQSR, fliEFGHIJKLMNOPQR, flhB, flhAFN, fliA* and *motCD*.

Most *P. aeruginosa* isolates express one of two types of flagella (A or B) based on the deduced amino acid sequences of the major structural gene *fliC*, encoding the flagellin filament [116]. *P. aeruginosa* flagella also have two potential types of glycosylation which occur in a strain-dependent fashion [117]. PAO1, LESB58, C3719 and PA14 all have type B flagellin and type b glycosylation alleles while the type A flagellin and type a glycosylation alleles present in PAHM4 have also been described in strains PAK, PA2192, NCGM2.S1 [117, 118], and are common in *P. aeruginosa* clinical isolates from patients with CF [119].

### Characterization of secondary metabolite capacity in PAHM4

Putative secondary metabolite clusters were identified with antiSMash [120]. PAHM4 was predicted to encode the clusters common to most *P. aeruginosa* strains. However, a PAHM4 gene cluster that is predicted to produce 3-oxobutanal is shared with M18 and PA-138224 but absent in other sequenced *P. aeruginosa* strains.

Acquisition of iron and zinc *in vivo* contributes greatly to *P. aeruginosa* metabolism and is crucial for the pathogenesis of this bacterium [121]. *P. aeruginosa* strains produce two siderophores, pyochelin and pyoverdine (reviewed by Cornelis [122]), and PAHM4 encodes gene clusters responsible for production of both compounds. The chromosomal region responsible for pyochelin has 98 % sequence identity to PAO1 [123]. The PAHM4 genome contains a type 3 pyoverdine cluster that is 99 % identical to the cluster identified in Serotype O13 strain *P. aeruginosa* ATCC O13 [124]. Despite the presence of an intact gene cluster, pyoverdine assays indicated that PAHM4 produces minimal pyoverdine (data not shown). The gene encoding sigma factor for pyoverdine expression, *pvdS*, has a nonsense mutation in PAHM4 and is likely the reason for the lack of pyoverdine production. While pyoverdine has been shown to be necessary for acute infections [125], gene expression data and sputum analysis from chronic CF infections have indicated that some strains lose the capacity to produce it [126, 127]. Assays for pyocyanin in overnight cultures of PAHM4 indicated a loss of pyocyanin production compared to PAO1 (data not shown).

### Microarray analysis provides a global view of adaptation of PAHM4 to chronic lung infection

**Fig. 10:**
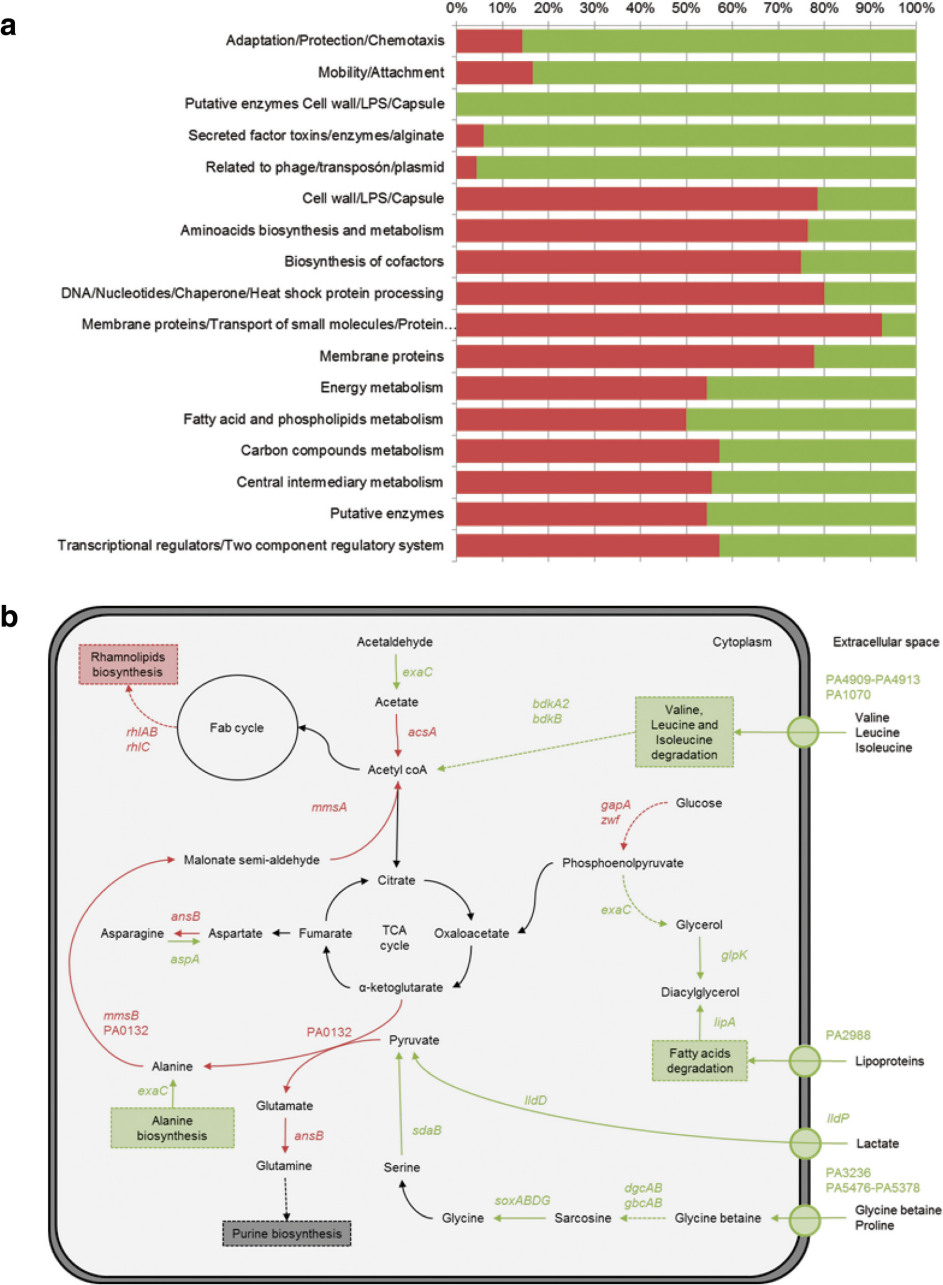
Functional analysis of differential gene expression. **a** Functional classification of the genes showing a differential expression in microarray analysis and based on PseudoCAP function class assignments [168]. The proportion of genes over-expressed in PAHM4 compared to PAO1 is indicated in green and the proportion of the genes down-regulated compared to PAO1 appears in red. Hypothetical, unclassified and unknown protein and non-coding RNA sequences were excluded from the analysis. **b** Adapted intermediary metabolism of PAHM4 to the bronchiectasis lung. Transcripts that were found to be significantly increased (green) or decreased (red) in PAHM4 compared to PAO1 are indicated in the pathway for central metabolism based on KEGG pathways [163].

Given the wide array of phenotypic changes seen in PAHM4, we compared the transcriptome of this strain to PAO1 when grown in standard laboratory conditions. A total of 164 genes were found to be significantly up-regulated and 168 down-regulated in PAHM4 compared to PAO1, with an overview of affected pathways in Fig. 10a, a detailed list of gene expression changes in Additional file 5: Table S5, and a heat map depicting the significantly regulated genes (Additional file 6: Data File 7). Recognizing that genomic differences between PAHM4 and PAO1 could confound our results, we assessed the coding sequence identity of all genes identified as highly differentially regulated between these strains. Of the down-regulated genes detected from the array data, 16.1 % were absent from the PAHM4 genome while, as anticipated, 100 % of up-regulated genes were encoded by PAHM4. Together, these results suggest that the genetic differences between PAHM4 and PAO1 had minimal impact on the array results.

Strikingly, many of the down‐regulated genes in PAHM4 are involved in virulence phenotypes, such as secreted factors, motility and attachment. This phenomenon is often observed in strains isolated from chronic lung infections in patients with CF [128]. With regards to PAHM4, down-regulation of virulence-related genes is likely either the result of negative selective pressure in the lung environment of patients with bronchiectasis and/or a consequence of the previously described gene loss in PAHM4.

In contrast, a significant number of the *P. aeruginosa* genes involved in metabolism (i.e. energy, fatty acid and amino acid metabolism) and transport (membrane proteins, transport of small molecules) were up‐regulated in PAHM4 compared to PAO1, possibly the result of selective pressure in the bronchiectasis lung.

The differences observed in the transcriptome of PAHM4 and PAO1 indicate that metabolism in this isolate is characterized by an increased turnover of amino acids (Fig. 10b). PAHM4 had increased levels of transcripts encoding branched amino-acid transporters (homologous to PA4909-PA4913 and PA1070) and degradation enzymes (*bdkA*2 and *bdkB*) compared to PAO1.

Transcript levels of the glycine betaine uptake system (homologous to PA3236 and PA5376-PA5378) and degradation pathway through the over expression of *gbdR*, responsible for controlling the expression of *gbcAB*, *dgcAB,* and *soxABDG* [129] were increased in PAHM4 compared to PAO1. Genes responsible for lipoprotein uptake (PA2988 homolog) and degradation (*lipA*) are also increased, along with pyruvate biosynthesis from serine and degradation of glycine and lactate. These data suggest that the metabolism of PAHM4 is adapted to preferentially use amino-acids and lipids, abundantly found in the lung secretions of patients with chronic lung infections [130], as sources of carbon, nitrogen and energy.

Transcriptome data indicate that the iron acquisition genes PA4834, PA4880, *lipA*, *lipH*, and PA1922, and zinc acquisition genes *np20* and *znuBC* [131] are up-regulated in PAHM4 compared to PAO1. Previous studies examining gene expression from *P. aeruginosa* clinical isolates in sputum compared to lab growth medium indicate that while PA4384 and PA1922 tended to be up-regulated, *lipA*, *lipH* and PA4880 were down-regulated [132]. The three zinc acquisition genes up-regulated in the current study were also up-regulated in CF sputum compared to LB in the study by Bielecki et al*.* (GEO accession GSE25945 [132]).

Metabolic adaptation of PAHM4 to the lung of a chronically infected bronchiectasis patient appears to differ from what has been observed in previously characterized CF isolates, which have been shown to have an increased arginine catabolism, up-regulation of branched amino-acids synthesis, unchanged levels of transcripts of enzymes involved in central metabolism and up-regulation of transcripts of enzymes involved in the TCA cycle [16]. These adaptations were not observed in PAHM4.

Defects in twitching and swarming motility described above may be a result of down-regulation of the rhamnolipid biosynthetic machinery (*rhlA*, *rhlB, rhlC, rhlI* and *rhlR*) (Additional file 5: Table S5), presumably due to a result of the loss of QS in this strain. Transcriptome analysis also indicated a decrease in expression of other motility-related genes in PAHM4 compared to PAO1 including chemotaxis (*ae2* and PA0177-PA0179), TFPa pilin (*pilA*), TFPb pilin, and flagellar biosynthesis machinery (*flgE, fliC,* PA1093, *fliD*, PA1095, PA1096). Rhamnolipid assays demonstrated a lack of rhamnolipid production by PAHM4 (data not shown). Similar results have been reported in hypermutator CF isolates [16].

### Metabolic modeling connects transcriptome analysis, genome sequence, and observed phenotypes

To understand the specific metabolic adaptations in the context of global metabolic function in PAHM4, transcriptome data was contextualized using a previously published genome-scale metabolic reconstruction of *P. aeruginosa,* iMO1086 [133, 134]. Accounting for the inter-dependent, metabolic functions of 1086 genes, this computational model can predict metabolic phenotypes relevant to chronic infection of the lung during steady state growth using flux balance analysis. Flux balance analysis (FBA) is used to predict the ability to grow by calculating the flux that is possible through an objective reaction (here representing compounds required for production of biomass) in a given environmental condition. This analytical method can also be used to predict the capacity for production of an array of virulence factors, connecting substrates to all intermediate pathways necessary for synthesis of the factors.

Computational predictions are improved with the integration of high-throughput profiling data such as gene expression into the model which captures the collection of genes that are active in a given condition [135]. Subsets of the differentially expressed genes identified in the transcriptome analysis were used to develop genome-scale metabolic models of PAO1 and PAHM4 using iMO1086. A total of 33 genes down-regulated in PAHM4 and 63 genes down-regulated in PAO1, as identified by the RankProd differential expression analysis, were accounted for in iMO1086. The models were constrained using GIMME, with an expression value cutoff to separate likely active and inactive genes. These analyses identified 113 likely inactive genes to incorporate into the conditional metabolic state of PAHM4 and 181 likely inactive genes to incorporate into the PAO1 metabolic state.

After applying these metabolic states to iMO1086 and performing FBA, several of the genes implemented as inactive in one or both models appeared necessary for growth. For example, though glyceraldehyde 3-phosphate dehydrogenase (*gapA* - PA3195) is significantly down-regulated in PAHM4 as indicated in Fig. 10b, inactivating this enzyme prevented *in silico* growth in rich media conditions. This prediction of the apparent essentiality of PA3195 suggests that while glyceraldehyde 3-phosphate dehydrogenase is expressed at low levels it is sufficient to enable growth and/or PAHM4 is expressing an alternate gene that enables glyceraldehyde 3-phosphate dehydrogenase activity. A search for potential alternate genes identified a probable glyceraldehyde 3-phosphate dehydrogenase (PA3005) that shows a small increase in expression in PAHM4 compared to PAO1. Additionally, erythrose 4-phosphate dehydrogenase (PA0551) is highly up-regulated in PAHM4 and may also be capable of low levels of glyceraldehyde 3-phosphate dehydrogenase activity, as has been shown for the *E. coli* homolog [136]. Including the catalytic activity of glyceraldehyde 3-phosphate dehydrogenase in the model restored *in silico* growth, demonstrating the potential ability of these two genes to compensate for the function of down-regulated PA3195 in PAHM4. Overall, the inactive genes that we reverted to active status in the metabolic states were few (PA3195, PA2023, PA4055, and PA4513) and some are likely artifacts of the conversion from relatively high and low gene expression values to binary on-off states. Additionally, iMO1086 is a large, well curated model, but it contains network gaps and incomplete implementation of the relationships between genes, proteins, and reactions that reflect the still-evolving genome annotation and understanding of *P. aeruginosa* metabolism for both strains studied here.

Using expression data to constrain iMO1086 into modPAHM4 and modPAO1 growing in rich media *in silico* enables the comparison of the conditional metabolic states of these strains. Essentiality of the active genes in each state was analyzed by preventing the function of the remaining genes one at a time and optimizing for growth. Failure to achieve biomass flux indicated that the deleted gene was essential to the model’s growth in that state. This analysis showed that an almost identical set of genes was essential to *in silico* growth of modPAO1 and modPAHM4 on rich media (152 and 151 genes per model, respectively, Additional file 5: Table S6). As the number of genes classified as essential is nearly identical between strains, it confirms that important central metabolic functions are enabled in both strains though their levels of activity may not be comparable, as is suggested by the *gapA* example discussed above. *P. aeruginosa* can utilize a wide variety of carbon sources; growth can be achieved through many different catabolic pathways in rich media despite an array of inactive genes. However, functional metabolic changes induced by the gene expression integration are apparent in more peripheral pathways related to virulence factor production and amino acid utilization.

**Fig. 11:**
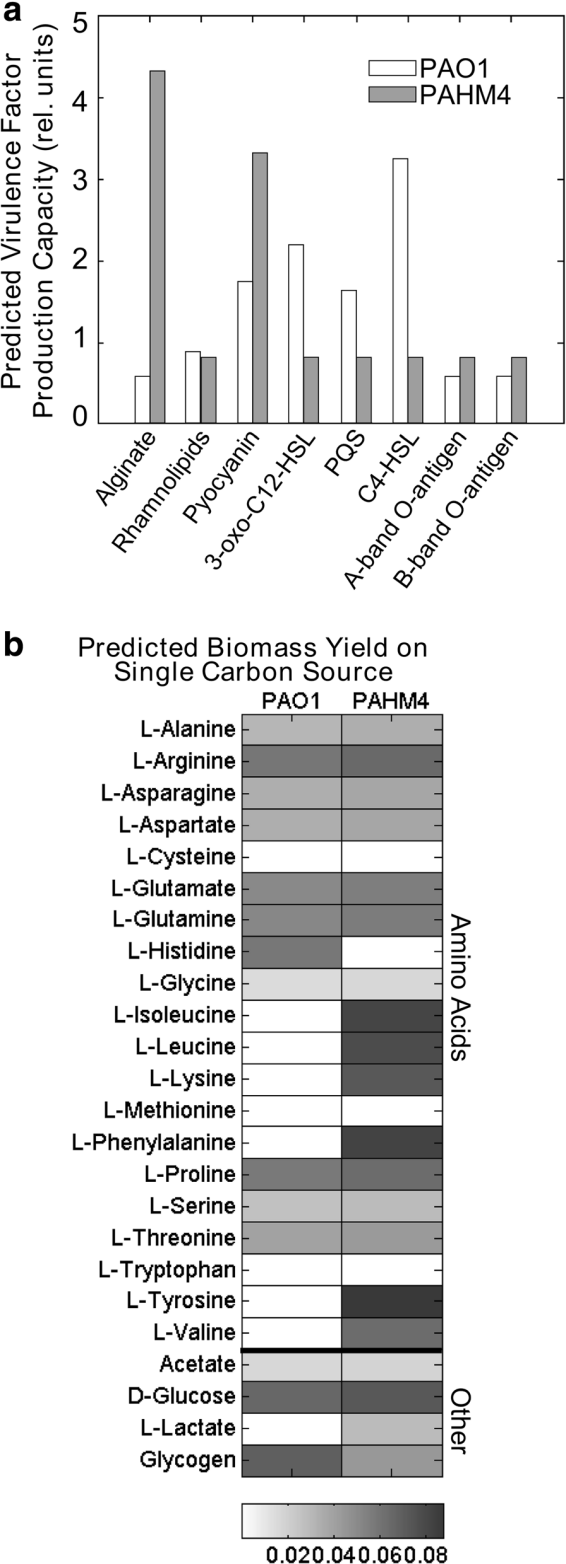
Expression-constrained metabolic modeling. **a** In silico model predictions of the potential capacity of PAO1 and PAHM4 to produce various virulence factors of interest when grown in rich medium. Capacity units correlate to the maximum flux through an objective reaction representing the production of each factor normalized by the max uptake flux through the limited carbon uptake reactions for the optimization. **b** In silico estimation of biomass yield during growth in minimal media conditions supplemented with a single carbon source (amino acids and a selection of sugars as shown). Presented values are flux through the optimized biomass function normalized by flux through the single carbon source uptake reaction

The models enable the comparison of the maximum theoretical production capacity of an array of virulence factors of PAHM4 and PAO1. For each virulence factor capacity prediction, the objective function was changed to a reaction enabling the maximal production of the virulence factor of interest [134, 137]. Fig. 11a shows that modPAHM4 has notably higher production capacity for the polysaccharide alginate and phenazine pyocyanin than modPAO1. While our previously described assays demonstrated a comparative lack of pyocyanin production in PAHM4, false positives for this trait were previously observed with an earlier version of the model and are hypothesized to be the result of regulatory changes within the bacterium that the model does not account for [137].

Contrastingly, modPAO1 has higher production capacity for QS molecules. LPS O-antigen production capacities remain similar for each strain, though further investigation of O-antigen genes as explained in a previous section suggests that a frame shift mutation is preventing O-antigen synthesis in PAHM4.

The models were then used to predict the utilization of carbon sources commonly present in the lung, such as amino acids. The *in silico* media was altered to only provide the models with a single carbon source in addition to the standard required salts and ions. Fig. 11b shows the difference in biomass yield on the carbon sources surveyed. It is important to note that the apparent inability of PAO1 to grow on certain substrates reflects our model implementation of down-regulated (but potentially active) genes from *in vitro* expression data as *in silico* inactivated genes, and PAO1 could catabolize many of the substrates given time to adapt its conditional expression profile in appropriate media. The integration of PAHM4 expression data shows that PAHM4 is currently better adapted to growth in the lung environment on a systemic level; modPAHM4 has a clear advantage over modPAO1 in its ability to catabolize amino acids as energy sources, particularly in the utilization of branched-chain amino acids. ModPAHM4 also shows higher capacity for phenylalanine degradation, and the utilization of L-lactate, both identified as important carbon sources in a study of cystic fibrosis lung sputum [138].

Thus, the expression-integrated comparative modeling predicts that PAHM4 has adapted to its available nutritional resources in the lung environment in addition to altering its virulence factor production profile. The model not only reflects the predisposition of PAHM4 towards amino acid catabolism, it evaluates all other pathways necessary for growth on amino acids and confirms on a genomic scale the heightened capacity for growth using these substrates. Additionally, the model quantifies the high capacity of PAHM4 for alginate and pyocyanin production while other virulence factor production capacities are lowered, expanding on a more functional level the insights provided by the transcriptome analysis and indicating important phenotypic changes relevant to virulence.

## Conclusions

In conclusion, we have presented the analysis of the phenotypic characteristics, genome, transcriptome, and metabolic model of a *P. aeruginosa* non-CF bronchiectasis isolate, showing that *P. aeruginosa* undergoes a similar process of adaptation in the lung of these patients compared to the lung of CF patients. However, we also identified a collection of genetic and phenotypic traits particular to this strain suggesting niche-specific differences and selection that occur in bronchiectasis. This distinction was typified by the *in vitro* infection phenotypes which differed from wound and burn isolates (PAO1 and PA14, respectively) as well as from acute and chronic CF isolates.

The results obtained in this study have advanced our understanding of *P. aeruginosa* virulence in the context of chronic bronchiectasis infections. However as PAHM4 is currently the only sequenced *P. aeruginosa* isolate from chronic bronchiectasis, evaluation of additional isolates would be useful for determining if the characteristics observed in PAHM4 are common to bronchiectasis isolates or strain specific. This additional characterization in turn will be vital to study the epidemiology and control of chronic infections in patients with bronchiectasis, and provide valuable insights for the identification of prospective therapeutic targets and intervention strategies.

## Methods

### Ethics statement

The clinical *P. aeruginosa* isolates described here, including PAHM4, originated from a study that was approved by the Research Committee of Hospital Son Espases (formerly Hospital Son Dureta).

Murine experiments were performed in strict accordance with the recommendations in the Guide for the Care and Use of Laboratory Animals of the National Institutes of Health. Protocols were approved by the Institutional Animal Care and Use Committee at the University of Virginia (Protocol number 2844). All efforts were made to minimize animal suffering during the course of these studies.

### Bacterial strains

Table 8 lists strain sources and serotypes for *P. aeruginosa* strains used in this analysis. *P. aeruginosa* strain PAHM4 was isolated in 2003 from the sputum of a 78 year old female patient at the Hospital Universitario Son Dureta (Palma de Mallorca, Spain). The patient was diagnosed with chronic diffuse bilateral bronchiectasis and was first documented with evidence of chronic respiratory infection by mucoid *P. aeruginosa* in 2000. The patient received multiple courses of antibiotics to treat the infection, including ciprofloxacin, cotrimoxazole, ceftazidime and several years of nebulized tobramycin.

**Table 8:** Origin of *P. aeruginosa* strains used in this analysis

*P. aeruginosa* strain	Disease isolate	Genome Status	Serotype^a^	Accession #	Reference
PAHM4	Chronic bronchiectasis	Draft	nt O13	AYSZ01000000	This study
PAO1	Wound	Complete	O5	NC_002516.2	[123]
PA14	Burn	Complete	O10	NC_008463.1	[171]
PA7	Wound	Complete	O12	NC_009656.1	[85]
PA2192	Cystic fibrosis	Complete	nt O1	NZ_AAKW00000000	[169]
LESB58	Cystic fibrosis	Complete	nt O6	NC_011770.1	[172]
NCMG2.S1	Urinary tract infection	Complete	O11	NC_017549.1	[173]
C3719	Cystic fibrosis	Complete	O3	NZ AAKV00000000	[169]
Serotype O13			O13		G.B. Pier

^a^ *nt* non-typable

### Bacterial growth and DNA preparation

*P. aeruginosa* strains were typically grown in LB (per L: 10 g tryptone, 5 g NaCl, 5 g yeast extract, solidified with 15 g Bacto-agar, as necessary) at 37 °C. Genomic DNA was purified by the method of Pospiech and Nuemann [139] modified with 30 min incubations.

### Adhesion and invasion assays

The human lung carcinoma cells, A549 (ATCC CCL185), derived from type-II pneumocytes were propagated in RPMI 1640 plus 1 % HEPES (hydroxyethyl piperazine ethane sulphonic acid), supplemented with 10 % fetal calf serum and 1 % penicillin-streptomycin (Sigma-Aldrich, St. Louis, MO, USA). Cells were cultured in 24-well tissue culture plates at 37 °C and 5 % CO_2_ until confluence was reached (~5 x 10^5^ cells per well).

Adhesion and invasion assays were performed as previously described [140]. Briefly, a bacterial suspension was prepared at 10^7^ colony-forming units (CFU)/mL in RPMI-HEPES and incubated for 1 h with the cell monolayers (multiplicity of infection (MOI) 20:1). The wells were washed with PBS and either lysed and plated to quantify adhesion or incubated with fresh medium containing gentamicin (100 μg/mL) to kill extracellular bacteria. After 1 h, an aliquot of the medium was plated to confirm the elimination of extracellular bacteria, and the gentamycin-containing medium was washed from the monolayer. The epithelial cells were lysed with a solution of 0.5 % Triton X-100 (Sigma-Aldrich) in PBS and intracellular bacteria were quantified by plating appropriate dilutions on LB agar plates. Experiments were performed in duplicate and the data were analyzed using an unpaired two-tailed *t*-test with GraphPad Prism 5.01.

### Alginate quantification

Alginate was quantified by carbazole determination of uronic contents adapted from Knutson et al. [141]. PAO1 and PAHM4 were grown on LB plates at 37 °C for 18 h. Colonies were resuspended in Ringer’s buffer (155 mM NaCl, 5 mM KCl, 2 mM CaCl_2_, 1 mM MgCl_2_, 2 mM Na_2_HPO_4_, 10 mM HEPES, 10 mM glucose) to an OD_420_ of 1. CFU were determined by plating the appropriate dilutions on LB agar plates. The suspension was vigorously vortexed for 15 min and centrifuged at 16,000 x g for 3 min. 500 μL of supernatant was incubated at 100 °C with 3 mL 0.025 M sodium tetraborate in sulfuric acid for 10 min. Then 100 μL of 0.125 % weight/volume carbazole (Sigma-Aldrich) in ethanol was added to the mixture and boiled for 15 min. The amount of alginate was measured spectrophotometrically at OD_530_ against a standard curve of glucuronolactone (Sigma-Aldrich) and expressed as micrograms of alginate per CFU.

### Macrophage phagocytosis assay

Bone marrow-derived macrophages were isolated from BALB/c mice as previously described [142]. Harvested macrophages were allowed to differentiate for 5 days at 37 °C in the presence of L-cell-conditioned medium. Macrophages were then trypsinized and 0.5 ×10^6^ cells were added to wells in 24-well plates and allowed to adhere for 2 h. Bacteria were grown at 37 °C in LB for 18 h before a suspension was prepared in minimum essential medium (MEM) and incubated with adherent macrophages for 15 min at an MOI of 500:1 (2.5 × 10^8^ CFU/well). After 15 min, cells were washed and lysed to quantify bacterial uptake or incubated with fresh medium containing gentamicin (100 μg/mL) to kill extracellular bacteria. After 15, 30, 60 and 90 min of incubation at 37 °C, cells were washed three times with PBS and lysed, as described above, to quantify intracellular bacterial survival by plating of appropriate dilution on LB agar. Experiments were performed in triplicate and the data were analyzed using an unpaired two-tailed *t*-test and the software GraphPad Prism 5.01.

### Lettuce infection assay

Lettuce leaves were infected as previously described [143]. Bacteria were grown at 37 °C in LB for 18 h and washed three times in sterile 10 mM MgSO_4_. Healthy-appearing leaves were obtained from commercially purchased romaine lettuce, washed with 0.1 % bleach and rinsed with sterile water. The midrib was infected with 10^6^ CFU and incubated at 25 °C for 48 h. The diameter of the soft rot area at the site of inoculation was measured. Experiments were performed in triplicate and the data were analyzed using an unpaired two-tailed *t*-test with GraphPad Prism 5.01.

### Biofilm quantification

Biofilm assays were based on previously described protocols [102]. Briefly, *P. aeruginosa* LB cultures were grown overnight and diluted to an OD_600_ of 0.1 in fresh LB unsupplemented or supplemented with 2 % glycerol and/or 100 μM FeCl_3_ [144, 145]. For each replicate, 150 μL of this suspension was added to a sterile 96 well flat bottom PVC microtitre plate and statically incubated for 48 h at 37 °C. After the growth period, plates were thoroughly rinsed with de-ionized water and stained with 165 μL 0.1 % crystal violet for 5 min. The plates were then rinsed to remove excess dye, allowed to air dry and the crystal violet was extracted with 175 μL of methanol. The OD_570_ of the methanol was measured and blanked against an uninoculated negative control. Samples were grown in duplicate with 5 technical replicates and the reported values are the average of the biological replicates with error bars representing the standard deviation.

### Analysis of secreted factors

Protease and elastase assays were done following protocols from Kessler et al. [146]. Briefly, cells were grown overnight in LB at 37 °C. The OD_600_ of the overnight cultures was determined before centrifugation to remove the cells. Supernatants were filtered through 0.22 μM filters and the filtrates were placed on ice until use. For the protease assay, a 0.3 % azocasein (Sigma-Aldrich) solution was made in buffer B (50 mM Tris–HCl, 0.5 mM CaCl_2_, pH 7.5). 1 mL of filtrate was added to 1 mL of azocasein solution which was then incubated at 37 °C for 15 min. After incubation, 500 μL of 10 % trichloroacetic acid (w/v) was added to each sample and briefly vortexed. Samples were centrifuged for 5 min at 18,000 × g at room temperature and the OD_400_ of the resulting supernatants was determined. The values reported are OD_400_/OD_600_. For the elastase assay, 400 μL of filtrate was added to a solution of 0.45 % elastin-congo red (w/v) (Sigma-Aldrich) resuspended in buffer B. The samples were then incubated shaking at 37 °C for 2 h. The reactions were stopped by adding 100 μL of 120 mM EDTA before centrifugation. Supernatants were removed and the OD_495_ was determined. Values reported are OD_495_/OD_600_. For protease and elastase assays, cultures were grown in triplicate and the reported values are the averages, with error bars representing the standard deviation. Probabilities were determined with Tukey’s multiple comparison test within GraphPad Prism.

### MutS characterization

*P. aeruginosa mutS* sequences were obtained from the Pseudomonas Genome Database (www.pseudomonas.com) [147] and as published by Warren and colleagues [11]. Sequence alignments were performed with ClustalW in CLC Main Workbench (CLC, Aarhus, Denmark, www.clcbio.com). The hypermutator phenotype was characterized by electroporating PAHM4 with plasmids harboring PAO1 wild-type *mutS* (pUCPMS) [40], *mutL* (pUCPML) [40] or *mutY* (pLM102) [148]. Transformants were selected on LB agar plates containing 250 μg/mL gentamycin (pUCPMS and pUCPML) or 200 μg/mL tetracycline (pLM102). To evaluate the complementation of the mutator phenotype, mutant frequencies and mutation rates were determined. Approximately 10^3^ cells from overnight cultures of either wild-type PAHM4 or PAHM4 harboring a plasmid were inoculated into each of ten 1 mL Müller-Hinton (MH) broth tubes and incubated for 24 h at 37 °C and 180 rpm. Serial dilutions were then plated on MH agar or on MH agar supplemented with 300 μg/mL rifampicin and incubated at 37 °C for 24 h (total CFUs) or 48 h (rifampicin-resistant mutants), respectively. Mutant frequencies (mutants per cell) were calculated by dividing the median number of mutants by the median number of total CFUs [40] and mutation rates (mutations per cell division) were estimated using fluctuation test (www.bio.upenn.edu/faculty/sniegowski/#software) [149].

### Motility assays

Swimming, swarming and twitching motility were determined as described previously [150]. Briefly, bacteria were grown overnight in LB and cells were transferred to swimming semi-solid agar medium (1 % tryptone, 0.5 % NaCl, and 0.3 % DNA grade agarose) or swarming semi-solid agar medium (0.5 % Nutrient broth, 0.5 % glucose, 0.5 % bacto-agar) using a sterile toothpick. The swimming and swarming zones were measured after 48 h incubation at 37 °C.

Twitching motility was measured in LB solidified with 1.2 % bacto-agar. Agar plates were inoculated with a toothpick through the media to the bottom of a Petri dish and incubated at 37 °C for 48 h. Following incubation, agar was removed and the plates were stained for 5 min with 0.5 % crystal violet. Swimming, swarming, and twitching assays were performed in triplicate.

### Antibiotic susceptibility testing

MICs of cefotaxime (CTX), ceftazidime (CAZ), cefepime (FEP), piperacillin (PIP), piperacillin-tazobactam (PTZ), aztreonam (ATM), imipenem (IMP), meropenem (MER), ciprofloxacin (CIP), gentamicin (GEN), tobramycin (TOB), amikacin (AMK) and colistin (COL) were determined on Müller-Hinton (MH) agar plates by Etest. Additionally, MICs of tetracycline (TET), azithromycin (AZT), ceftolozane (CTZ) and carbenicillin (CAR) were determined by broth microdilution in MH. All MICs were determined in duplicate experiments for strains PAHM4 and wild-type reference strain PAO1. When available, the breakpoints recommended by the Clinical Laboratory Standards Institute (CLSI) [151] were used to assign the corresponding clinical susceptibility categories: susceptible, intermediate, or resistant.

### Expression of resistance genes

The levels of expression of *ampC*, *creD*, *mexB*, *mexD*, *mexY*, and *mexF* were determined by RT-qPCR following previously described protocols [45, 152]. Briefly, strains were grown to an OD_600_ of 1 in 10 mL of LB at 37 °C and shaking at 180 rpm. Total RNA was isolated from cell pellets using an RNeasy minikit (Qiagen, Carlsbad CA). RNA was eluted with RNAse-free water and treated with 2 U of Turbo DNase (Ambion, Austin TX) for 30 min at 37 °C to remove contaminating DNA. The reaction was stopped by the addition of 5 μL of DNase inactivation reagent. 50 ng of purified RNA was used for one-step reverse transcription and real-time PCR amplification using a QuantiTect SYBR green RT-PCR kit (Qiagen, Carlsbad CA) and a SmartCycler II system (Cepheid, Sunnyvale CA). Previously described primers and conditions [45] were used for amplification of *ampC*, *creD*, *mexB*, *mexD*, *mexY*, *mexF*, with *rpsL* as a reference gene [153]. In all cases, the mean values of relative mRNA expression obtained in at least three independent duplicate experiments were considered. For *ampC* and *creD* induction experiments, cultures were incubated in the presence of 50 μg/mL cefoxitin.

### Genome sequencing, assembly, closure and annotation

Genome sequencing and *de novo* assembly was performed by Otogenetics (Athens, GA). Briefly, a 175 bp mate pair library was produced and used to generate approximately 10 million 110 bp reads using the Illumina HiSeq platform. *de novo* assembly was accomplished using SOAP DeNovo [154]. The preliminary *de novo* assembly resulted in 732 contigs and scaffolds, with an average length of 8,791 bp, a median length of 127 bp, and a total predicted genome size of 6,426,282 bp. The resulting N50 and N90 were 64,165 bp and 17,120 bp, respectively. This assembly was submitted to GenBank (AYSZ00000000), and was annotated with the Rapid Annotation using Subsystem Technology (RAST) server [155]. The assembly was improved by alignment of PAHM4 contigs with completed *P. aeruginosa* genomes and combining them where overlaps were present, as described in detail in the supplemental methods contained within Additional file 7: Data File 4. The projected genome size is 6,381,186 bp and the final assembly, included as Additional file 1: Data File 1, has an N50 of 203,730 bp, an N90 of 71,127 bp, and was used for all subsequent analyses. The annotation of this assembly is included as Additional file 2: Data File 2.

### Comparative genomics

Genome characteristics of *P. aeruginosa* strains used for bioinformatic comparisons to PAHM4 are listed in Table 4. Unless otherwise noted, data were collected from the RAST Seed Viewer [155]. Multi-locus sequence type (MLST) analysis was performed at the *Pseudomonas aeruginosa* MLST website (pubmlst.org/paeruginosa/) [48].

PAHM4-specific chromosome regions and *P. aeruginosa-*pan-genome regions absent in PAHM4 were identified using PanSeq (lfz.corefacility.ca/panseq/) [50]. PanSeq was run with default parameters, except for minimal fragment size which was set at 300 bp and the analyzed PAHM4 genome was limited to contigs and scaffolds larger than 300 bp.

### Secondary metabolite prediction

Secondary metabolite prediction was performed using antiSMash (antismash.secondarymetabolites.org) [120]. The PAHM4 contigs were concatenated based on the alignment with PA14 and all *P. aeruginosa* strains were analyzed using default settings.

### Prophage characterization

Putative prophage were identified from *P. aeruginosa* genomes using ProphageFinder (131.210.201.64/~phage/ProphageFinder.php) [56] and PHAST (phast.wishartlab.com) [55]. ProphageFinder was used to aggressively detect prophage remnants in the genome and was run with default settings, except for hit spacing, which was set at 6,000 bp, and tRNA scan, which was enabled. PHAST was used to conservatively identify intact or mostly intact prophage within the genome.

### Preparation and analysis of lipopolysaccharide (LPS) and lipid A

*P. aeruginosa* strains were grown overnight at 37 °C in LB. LPS preparation used 1 mL of cultures adjusted to an OD_600_ of 0.5 by a method modified from Davis and Goldberg [156]. Briefly, cell pellets were generated, resuspended in 200 μL of 1X sodium dodecyl sulfate (SDS) buffer (0.1 M Tris–HCl pH 6.8, 2 % β-mercaptoethanol (w/v), 2 % SDS (w/v), 10 % glycerol (w/v)) and boiled for 15 min. Upon cooling, DNAse and RNAse were added to final concentrations of 10 μg/mL each, and the samples were incubated at 37 °C for 30 min. Proteinase K was then added to a final concentration of 10 μg/mL and the samples further incubated for 60 min at 59 °C. 200 μL of 2X SDS buffer was added to the samples, and 10 μL was run on a 12 % polyacrylamide gel and transferred to nitrocellulose using a Trans-Blot Cell (Biorad, Hercules CA). The blots were analyzed using polyclonal serogroup O13 antiserum (Accurate Chemical & Scientific, Westbury NA) or common antigen-specific monoclonal antibody (MAb) N1F10 (J.S. Lam, University of Guelph, Guelph, Ontario, Canada). The secondary antibodies were goat anti-rabbit immunoglobulin G coupled to IR dye 680 (Molecular Probes, Eugene OR) or anti-mouse immunoglobulin G coupled to horseradish peroxidase (Sigma-Aldrich), respectively. LPS was directly visualized by staining a 12 % polyacrylamide gel with ProQ Emerald 300 Lipopolysaccharide Gel Stain (Molecular Probes, Eugene OR).

Lipid A samples were isolated from 25 mL overnight cultures grown in LB supplemented with 1 mM MgCl_2_. Cell pellets corresponding to 5 mL of this culture were resuspended in 400 μL isobutyric acid/1 M ammonium hydroxide in a 5:3 vol:vol ratio. Samples were boiled for 1 h with occasional vortexing. Tubes were cooled on ice, and then centrifuged at 200 x g for 15 min. The supernatant was transferred to a new test tube and diluted with 1.5 volumes of water followed by overnight lyophilization. Dried samples were washed twice with 1 mL methanol. Lipid A was extracted in 100–200 μL chloroform/methanol/water 3:1.5:0.25 (vol:vol:vol). A final centrifugation step was carried out at 2,000 × g for 1 min to pellet debris. Lipid A isolates were analyzed using negative-ion matrix-assisted laser desorption ionization time of flight (MALDI-TOF) mass spectrometry (MS) experiments as previously described [157]. Norharmane MALDI matrix in chloroform/methanol 2:1 (vol:vol) was applied to the sample plate followed by 1 μL of purified lipid A. Experiments were performed using a Bruker Autoflex MALDI-TOF mass spectrometer (Bruker Daltonics Inc., Billerica, MA). Each spectrum was an average of 300 shots. ESI tuning mix (Agilent, Palo Alto, CA) was used as a calibration standard.

### Quorum sensing (QS) analysis

The production of known *P. aeruginosa* autoinducers C4-acyl homoserine lactone (C4-HSL) and C12-acyl homoserine lactone (C12-HSL) were assayed using published protocols with the *P. aeruginosa rhl* reporter pECP65.1 and *las* reporters pJN105 and pSC11, respectively [79, 80]. Triplicate cultures of *P. aeruginosa* PAO1 and PAHM4 were grown overnight at 37 °C with shaking in LB with 200 mM MOPS, pH 7, and supernatants were filter sterilized and used for β-galactosidase assays measuring QS-induced reporter activity. β-galactosidase assays were performed by standard protocols [158].

### Total RNA isolation and microarray analysis

*P. aeruginosa* strains PAO1 and PAHM4 were grown in triplicate at 37 °C in 50 mL of LB to an OD_600_ of 3 and stabilized with RNA protect (Qiagen, Carlsbad CA). The PAO1 37 °C array data has been recently described [159]. RNA was processed as described previously [160]. Briefly, RNA was extracted using RNeasy mini kit (Qiagen, Carlsbad CA), and samples obtained from three different cultures were pooled and amplified following the MessageAmp II Bacteria procedure (Ambion, Austin TX). 1 μg of RNA was polyadenylated and the complementary strand was synthesized by reverse transcription primed with T7-oligo-dT. RNA was removed by treatment with RNase H and second strand synthesis was accomplished with DNA polymerase. Antisense amplified RNA (aRNA) was transcribed from this template using T7 RNA polymerase with biotinylated dUTP and dCTP. aRNA quality, concentration and possible degradation was assessed on a 2100 BioAnalyzer (Agilent Technologies, Santa Clara CA).

aRNA was fragmented using 5x fragmentation buffer and the total amount of fragmented biotinylated aRNA used per chip was 6.5 μg. Biotinylated aRNA was then spotted in triplicate on *P. aeruginosa* GeneChips® (Affymetrix, Santa Clara CA) following the procedure established by the manufacturer. The hybridization and washing steps were performed in the Affymetrix Array facility at the Helmoltz Center for Infection Research (HZI) in Braunschweig, Germany.

The analyses of the arrays were performed using Bioconductor microarray analysis suite [161]. The quality of all chips was assessed by fitting a linear model to the probe level data using the fitPLM function from the affyPLM package. Expression values were computed using the Robust Multichip Average algorithm. Differentially expressed genes were identified using the Rank Products algorithm [162]. The value of 0.05 was accepted as a cut-off for pfp.

Transcriptome data were mapped to metabolic pathways at the Kyoto Encyclopedia of Genes and Genomes (www.genome.jp/kegg) [163].

### Metabolic modeling

Expression data for metabolic genes was contextualized using the well-curated genome scale metabolic reconstruction of *P. aeruginosa* PAO1, referred to as iMO1086, which captures the relationship between 1021 metabolites, 1031 reactions, and 1086 genes using a stoichiometric matrix connecting metabolites to reactions and gene-protein-reaction relationships (GPRs) [134].

Significantly down-regulated genes were identified by applying the Rank Products algorithm to the *P. aeruginosa* PAO1 and PAHM4 transcriptomics data. The GIMME (Gene Inactivity Moderated by Metabolism and Expression) algorithm [164] included in TIGER (Toolbox for Integrating Genome-scale Metabolism, Expression, and Regulation), a Matlab toolbox designed to complement the abilities of the COnstraint-Based Reconstruction and Analysis (COBRA) toolbox for performing FBA [165], was used to integrate expression data with the metabolic network reconstruction. For the GIMME analysis, an expression value threshold was defined as the minimum expression value of all array data plus 0.5 % of the full expression value range. This data integration resulted in two models representative of PAO1 and PAHM4. Biomass yield, gene essentiality, virulence factor production capacity and conditional carbon source utilization were analyzed for each model using FBA as has been previously described [166]. All analyses were conducted using COBRA Toolbox 2.0.5 [167] and TIGER Toolbox 1.3.0 in MATLAB.

### Accession numbers

The following *P. aeruginosa* genome sequences were used for comparisons throughout this manuscript: PAO1[GenBank:NC_002516.2], PA14 [GenBank:NC_008463.1], PA7 [GenBank:NC_009656.1], PA2192 [GenBank:NZ_AAKW00000000], LESB58 [GenBank:NC_011770.1], NCGM2.S1] [GenBank:NC_017549.1], C3719 [GenBank:NZ AAKV00000000], M18 [GenBank:NC_017548.1].

### Bacterial strain and data availability

The *P. aeruginosa* strain PAHM4 has been deposited in the repository of the American Type Culture Collection under the reference number BAA-2530. The Whole Genome Shotgun project has been deposited at DDBJ/EMBL/GenBank under the accession AYSZ00000000. The version described in this paper is version AYSZ01000000. Microarray data have been deposited to the National Center for Biotechnology Information’s Gene Expression Omnibus (GEO) and are accessible through GEO series accession number GSE40461.

### Additional data files

The following additional data files are available with the online version of this paper. Additional files 1 and 2 are FASTA-format files containing the manually assembled genome sequence of PAHM4 and RAST annotations of predicted ORFs of PAHM4 used for analysis in this paper, respectively. Additional file 7 contains supplemental methods including a description of the manual assembly procedure used to generate the data in Additional file 1, and descriptions of how Additional file files 3, 4, and 6 were generated, and the accompanying figure legends. Additional file 5 contains Supplemental Tables S1-S6. These tables contain, respectively, Novel DNA regions in PAHM4, Conserved DNA regions absent in PAHM4, Prophage contents of PAHM4, VgrG and Hcp complement of PAHM4, Up- and down-regulated genes from PAHM4 microarray analysis, and genes identified as essential in modPAO1 and modPAHM4. Three supplemental images are included as Additional file 3 (BRIG plot of genomes compared to PAO1), 6 (BRIG plot of genomes compared to PAHM4), and 7 (heat map of significant array results).

## Additional files

Additional file 1: (FA 6441 kb) Additional file 2: (FA 2008 kb) Additional file 3: (PNG 2085 kb) Additional file 4: (PNG 2553 kb) Additional file 5: (XLSX 63 kb) Additional file 6: (TIFF 17230 kb) Additional file 7: (DOCX 13 kb)

## Abbreviations

CF: Cystic fibrosis
COPD: Chronic obstructive pulmonary disease
LB: Lysogeny broth
LPS: Lipopolysaccharide
RT-qPCR: Reverse transcription quantitative PCR
T6SS: Type 6 secretion system
QS: Quorum sensing
MIC: Minimum inhibitory concentration
AHL: Acyl homoserine lactone
T2SS: Type 2 secretion system
HSI: Hcp1 secretion island
TFP: Type iv pili
CUP: Chaperone usher pathway fimbriae
FBA: Flux balance analysis
CTX: Cefotaxime
CAZ: Ceftazidime
FEP: Cefepime
PIP: Piperacillin
PTZ: Piperacillin-tazobactam
ATM: Aztreonam
IMP: Imipenem
MER: Meropenem
CIP: Ciprofloxacin
GEN: Gentamicin
TOB: Tobramycin
AMK: Amikacin
COL: Colistin
TET: Tetracycline
AZT: Azithromycin
CTZ: Ceftolozane
CAR: Carbenicillin
