# Supplementary figures and images for: Genotypic and phenotypic analyses of a Pseudomonas aeruginosa chronic bronchiectasis isolate reveal differences from cystic fibrosis and laboratory strains
Source: BMC Genomics. 2015 Oct 30;16:883. doi: 10.1186/s12864-015-2069-0 (PMC4628258; doi:10.1186/s12864-015-2069-0)

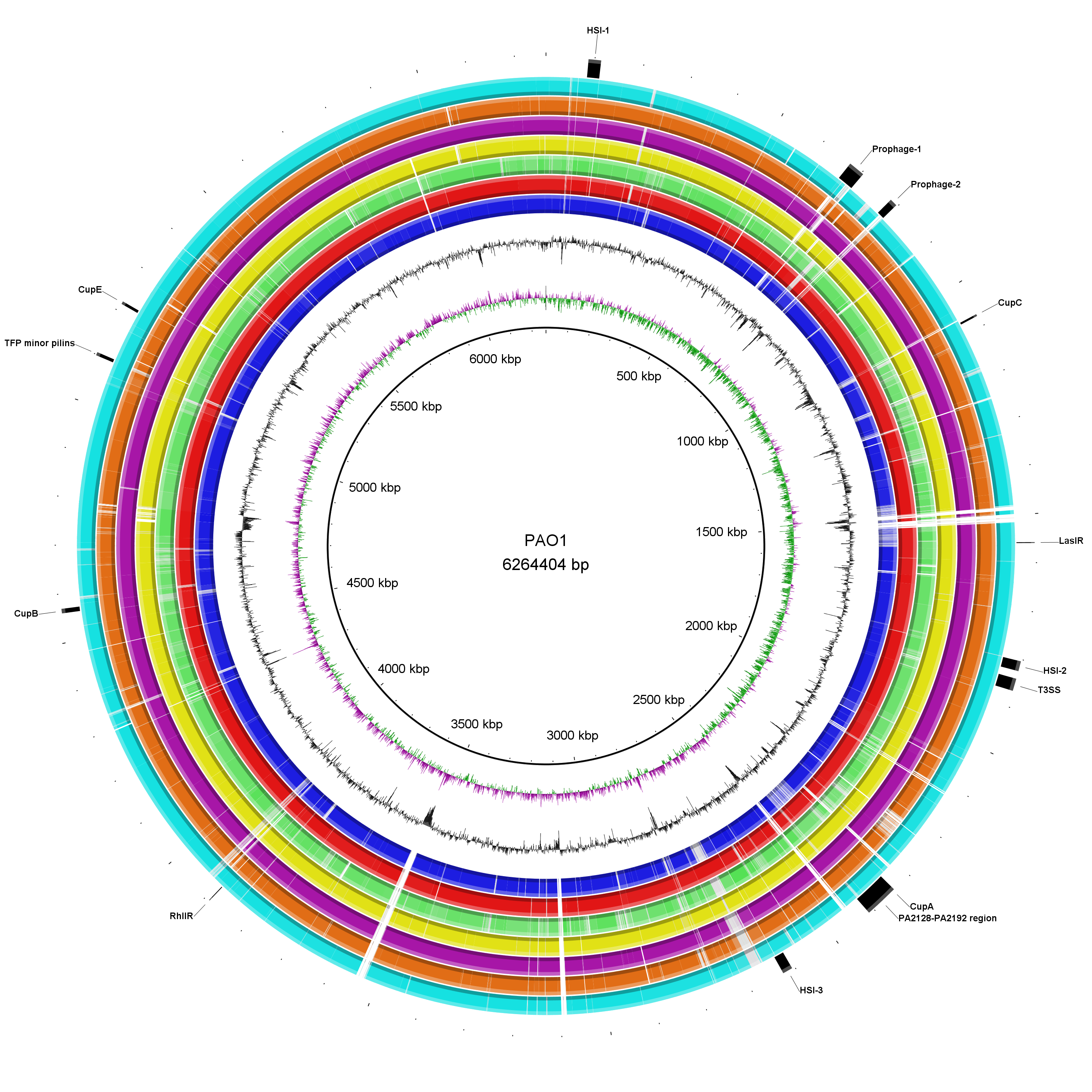

Supplement: Additional file 3: — (PNG 2085 kb) [file 12864_2015_2069_MOESM3_ESM.png]

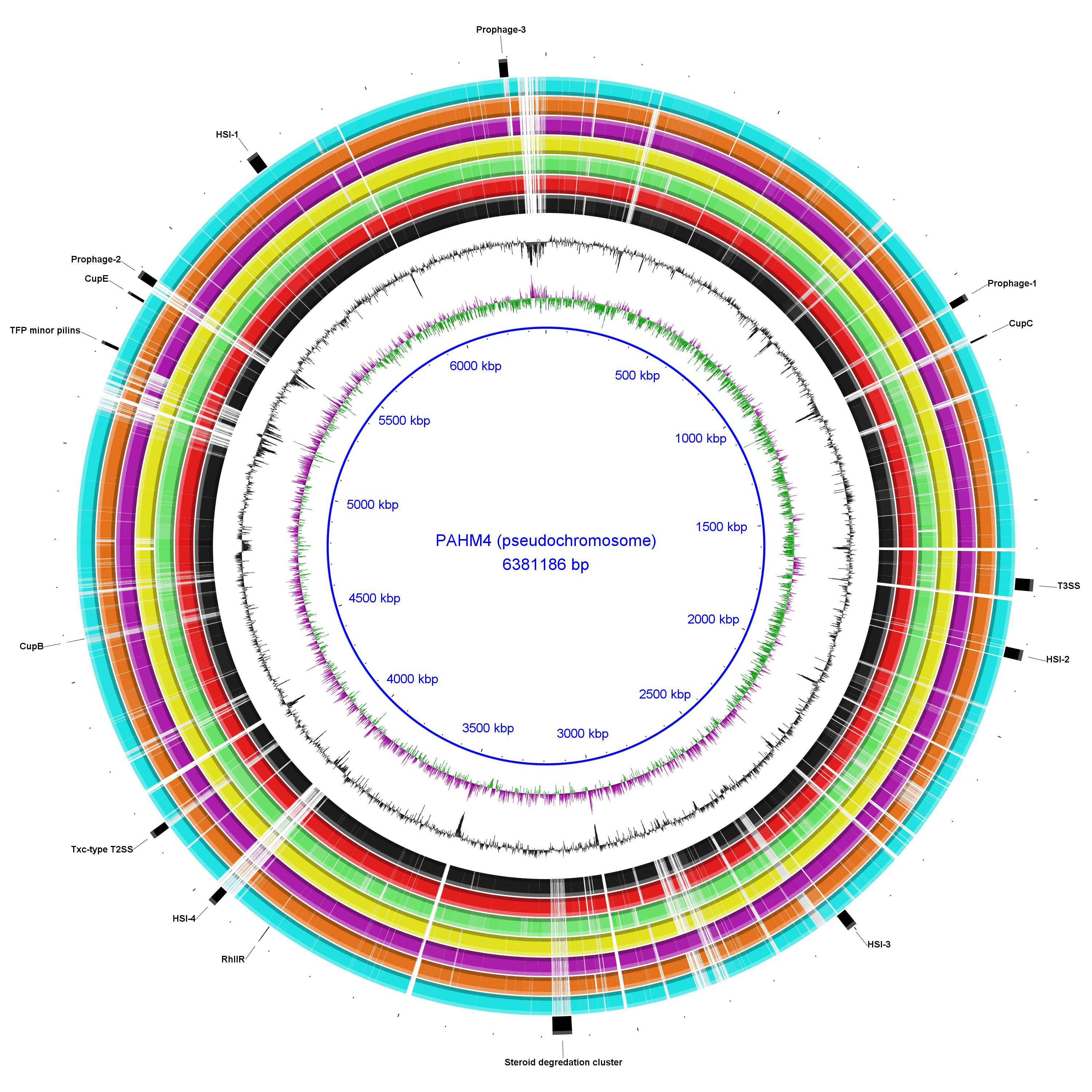

Supplement: Additional file 4: — (PNG 2553 kb) [file 12864_2015_2069_MOESM4_ESM.png]
