# Supplementary material for: Genotypic and phenotypic analyses of a Pseudomonas aeruginosa chronic bronchiectasis isolate reveal differences from cystic fibrosis and laboratory strains
Source: BMC Genomics. 2015 Oct 30;16:883. doi: 10.1186/s12864-015-2069-0 (PMC4628258; doi:10.1186/s12864-015-2069-0)
Supplement: Additional file 7: — (DOCX 13 kb) [file 12864_2015_2069_MOESM7_ESM.docx]

Supplemental methods

Manual assembly of PAHM4 contigs

De novo assembly of the *Pseudomonas aeruginosa* PAHM4 sequences resulted in 732 contigs and scaffolds with an N50 and N90 of 64,165 bp and 17,120 bp, respectively. A BLASTN database was generated within CLC Main Workbench (CLC, Aarhus, Denmark, www.clcbio.com) consisting of these contigs and scaffolds. All 732 individual sequences were then used as queries against this database and overlapping regions were identified. In instances where there were reciprocal best hits indicating identical overlaps, sequences were joined. If the resulting junction sequence was present in a BLASTN against a database of *P. aeruginosa* sequences the shorter parent sequences were placed with the joined sequence. After several rounds of this, the resulting sequences were annotated with RAST (46) and aligned with *P. aeruginosa* PAO1 and PA14.

The gaps were bioinformatically addressed in 2 ways. PAHM4 sequences bordering gaps were queried against a new database consisting of the newly assembled contigs and older unassembled contigs to identify overlaps to attempt to bridge the gaps. The PAO1 or PA14 sequence present in the gaps was also queried against the PAHM4 database to verify that the sequence was not present elsewhere due to either transposition, rearrangement, or an assembly artifact.

As additional overlaps were identified the process was repeated, and successive rounds of BLASTN, annotation and alignment were performed until the total contig count was reduced to less than 100.

The sequence at NCBI (Accession # AYSZ00000000) is the original output from Otogenetics (Athens, GA) while the final manually assembled sequence is attached as Supplemental File 1. Annotated peptide sequences are included as Supplemental File 2. For all analyses in the manuscript, the manually assembled genome was used.

## Generation of circular genome comparisons

BRIG (Alikhan *et al*, 2011) was used to generate circular genome comparisons. The program was run on a 64 bit Windows 7 PC running 64 bit Java version “1.8.0_51” with the command line “java -Xmx3500M -jar BRIG.jar”. Image settings were changed to a canvas size of 3000 x 3000 pixels. The color intensity defaults were changed from 70% identity maximum and 50% identity minimum to 90% maximum and 70% minimum. The PAHM4 sequence used for this analysis was a pseudochromosome generated by joining the assembly sequences in Additional Data File 1 in order based on alignment against the PA14 genome. The features track was manually added from information in the genome annotation of PAO1 or BLAST results against the PAHM4 pseudochromosome.

## Visualization of array results

Gene expression data were clustered by single linkage using Euclidian distance. Analysis was performed using CLC Mainworkbench 7.6.2 to generate a heat map.

# Supplemental figure legend

**Additional Data File 5: Circular comparison of the PAO1 chromosome to the PAHM4 pseudochromosome and other *P. aeruginosa* chromosomes.**

Brig was used to generate a circular multi-genome comparison. The inner-most ring represents G+C skew, with purple indicating a negative deviation from the average G+C and green indicating a positive deviation from the average. The next ring represents the average G+C content. The next 7 rings represent BLAST results of PAO1 again the following genomes: PAHM4 (blue), PA14 (red), PA7 (green), PA2192 (yellow), LESB58 (purple), C3719 (orange), and NCGM2.S1 (aqua). Gaps in the colored rings indicate that no sequence of at least 50% identity is present. The outermost ring indicates the location of several features of interest discussed in the main manuscript.

**Additional Data File 6: Circular comparison of the PAHM4 pseudochromosome to the PAO1 and other *P. aeruginosa* chromosomes.**

The same analysis was performed as for Additional Data File 5, however the reference genome was the PAHM4 pseudochromosome and the first genome ring is PAO1 (black). All other analysis details are the same between the two images.

**Additional Data File 7: Heat map analysis of PAO1 and PAHM4 gene expression.**

Hierarchical clustering of PAO1 and PAHM4 gene expression data obtained by microarray analysis. Genes with high expression values appear in red and genes with low expression value in blue. Hierarchical clustering of the samples if shown under the heat map. Analysis was performed using CLC Mainworkbench 7.6.
